# The human olfactory transcriptome

**DOI:** 10.1186/s12864-016-2960-3

**Published:** 2016-08-11

**Authors:** Tsviya Olender, Ifat Keydar, Jayant M. Pinto, Pavlo Tatarskyy, Anna Alkelai, Ming-Shan Chien, Simon Fishilevich, Diego Restrepo, Hiroaki Matsunami, Yoav Gilad, Doron Lancet

**Affiliations:** 1 Department of Molecular Genetics, Weizmann Institute of Science, Rehovot, Israel; 2 Section of Otolaryngology-Head and Neck Surgery, University of Chicago, Chicago, IL USA; 3 Department of Molecular Genetics and Microbiology, Department of Neurobiology, Duke Institute for Brain Sciences, Duke University Medical Center, Durham, NC USA; 4 Department of Cell and Developmental Biology, Neuroscience Program, and Rocky Mountain Taste and Smell Center, University of Colorado School of Medicine, Aurora, CO USA; 5 Department of Human Genetics, University of Chicago, Chicago, IL USA

**Keywords:** Olfaction, RNAseq, Olfactory epithelium, Olfactory receptor

## Abstract

**Background:**

Olfaction is a versatile sensory mechanism for detecting thousands of volatile odorants. Although molecular basis of odorant signaling is relatively well understood considerable gaps remain in the complete charting of all relevant gene products. To address this challenge, we applied RNAseq to four well-characterized human olfactory epithelial samples and compared the results to novel and published mouse olfactory epithelium as well as 16 human control tissues.

**Results:**

We identified 194 non-olfactory receptor (OR) genes that are overexpressed in human olfactory tissues vs. controls. The highest overexpression is seen for lipocalins and bactericidal/permeability-increasing (BPI)-fold proteins, which in other species include secreted odorant carriers. Mouse-human discordance in orthologous lipocalin expression suggests different mammalian evolutionary paths in this family.

Of the overexpressed genes 36 have documented olfactory function while for 158 there is little or no previous such functional evidence. The latter group includes GPCRs, neuropeptides, solute carriers, transcription factors and biotransformation enzymes. Many of them may be indirectly implicated in sensory function, and ~70 % are over expressed also in mouse olfactory epithelium, corroborating their olfactory role.

Nearly 90 % of the intact OR repertoire, and ~60 % of the OR pseudogenes are expressed in the olfactory epithelium, with the latter showing a 3-fold lower expression. ORs transcription levels show a 1000-fold inter-paralog variation, as well as significant inter-individual differences. We assembled 160 transcripts representing 100 intact OR genes. These include 1–4 short 5’ non-coding exons with considerable alternative splicing and long last exons that contain the coding region and 3’ untranslated region of highly variable length. Notably, we identified 10 ORs with an intact open reading frame but with seemingly non-functional transcripts, suggesting a yet unreported OR pseudogenization mechanism. Analysis of the OR upstream regions indicated an enrichment of the homeobox family transcription factor binding sites and a consensus localization of a specific transcription factor binding site subfamily (Olf/EBF).

**Conclusions:**

We provide an overview of expression levels of ORs and auxiliary genes in human olfactory epithelium. This forms a transcriptomic view of the entire OR repertoire, and reveals a large number of over-expressed uncharacterized human non-receptor genes, providing a platform for future discovery.

**Electronic supplementary material:**

The online version of this article (doi:10.1186/s12864-016-2960-3) contains supplementary material, which is available to authorized users.

## Background

Olfaction, the sense of smell, is a versatile and sensitive mechanism for detecting and discriminating thousands of volatile odorants. Olfactory recognition is mediated by a large repertoire of olfactory receptors (ORs), which activate a G-protein-mediated transduction cascade [1–4]. The ORs are expressed on the ciliated dendrites of olfactory sensory neurons located in the olfactory epithelium. Each sensory neurons expresses a single allele of a single OR gene locus, to ensure a distinct pattern of neuronal activation for every odorant [1, 2].

Olfactory epithelium, the tissue analyzed here, is heterogonous, containing besides the sensory neurons also epithelial supporting cells and progenitor basal cells, as well as sub-epithelial Bowman’s glands cells that secrete the mucus within which olfactory cilia reside, microvillar cells, and fingerlike microvilli cells [5]. While in mouse this tissue is readily available, the human counterpart is harder to obtain, due to difficulties in dissection and in defining the exact anatomically boundaries [6]. This explains the relative lack of transcriptome information about human olfactory genes. Overcoming these difficulties, we provide here a whole genome expression view of the human olfactory tissue.

Olfactory transduction can be divided into ligand binding, signal generation and signal termination. The end result is triggering of action potentials conducted along the axon to the olfactory bulb. A large number of proteins take part in such processes, as well as in the development and maintenance of the relevant cellular components [7–9]. Such gene products have been termed “auxiliary”, as portrayed in a digital compendium, GOSdb database (http://genome.weizmann.ac.il/GOSdb/, [10]).

Not all olfactory auxiliary genes have been identified, and most of them have never been studied in humans. Physiological differences among mammalian species may be accompanied with differences at the signal transduction level as well. For example guanylate cyclase 2D (*Gucy2d, GC-D*) gene, which is expressed in specific subset of OSNs and involved in CO_2_ detection [4, 11], was shown to be a pseudogene in humans, and signaling through this system appears to have been lost during primate evolution [12]. Here, we employ a broad transcriptome analysis to help fill some of these knowledge gaps, pertaining to olfactory auxiliary genes.

The human genome contains 857 OR genes, of which 391 are intact and 466 are pseudogenes with disrupting mutations in the open reading frame [13]. The repertoire of OR coding regions was deciphered mainly by computational genome data-mining [10, 14–16]. This information is reflected in genomic databases of both human and mouse, where most OR genes are portrayed with a partial gene structure that depicts only the coding region. The general reported ORs gene structure shows a single exon encompassing both a ~960 bp coding region and a 3’ untranslated region (3’UTR), with additional 5’ UTR short exons separated by long introns [17–20]. In mouse, cDNA sequencing and RNAseq, including single cell RNAseq of olfactory sensory neurons, have provided considerable specific information on OR gene structure [18, 20–25]. In contrast, information of the human OR gene structure is available only for a limited number of genes. Here we considerably expand this information.

The current study describes the transcriptome analysis of four human olfactory epithelial samples. We identified a set of 196 olfactory over expressed genes, composed of genes with known olfactory functions as well as novel olfactory candidates. This provides clues to a large number of uncharted genes which might have a role in the olfactory epithelium, including chemosensory function.

For OR genes we observed large variation in the expression intensity, as well as inter-individual differences in expression. We were also able to assemble the complete gene structure of 100 OR intact genes, providing a fresh general view of the encoded human OR transcripts.

## Results

### Differential expression

**Fig. 1:**
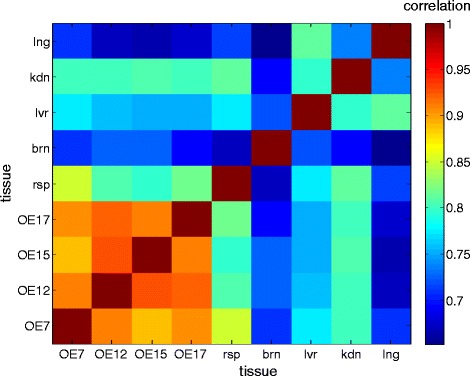
Expression correlation of olfactory and other tissues. Pearson correlation of human olfactory epithelium and a selected set of control tissues. Correlation was calculated for each tissue pair based on vectors with the logarithmic FPKM value of 16,288 genes. Correlation values are on average 0.91 ± 0.0 among the olfactory epithelium tissues and 0.74 ± 0.1 between the olfactory epithelium tissues to the controls. Tissue abbreviations: OE, human olfactory epithelium; rsp, respiratory; brn, brain; lvr, liver; kdn, kidney; lng, lung

We obtained epithelial samples from the nasal cavity of 14 human subjects and three autopsies. Four of them were identified by gene marker analysis as relatively high quality olfactory epithelium, and were selected for RNA sequencing (see Methods, Additional file 1: Table S1, Figure S1, Additional file 2: Table S2). The olfactory epithelial tissues portrayed a unique overall gene expression signature as compared to 16 control tissues from the illumina Body Map project (Fig. 1). For comparisons, we analyzed mouse olfactory epithelial RNAseq data from different sources (Additional file 2: Table S2), including a preparation of isolated sensory neurons. The latter provided specific information about the role of certain genes in this class of neuronal cells within olfactory epithelium. Although the mouse RNAseq came from different mouse strains, sex and age, the correlation values between the different strains as well as within strains was high and significant (MOE1-MOE2 0.945, MOE1-MOE3 0.947, MOE2-MOE3 0.978, Pearson).

**Fig. 2:**
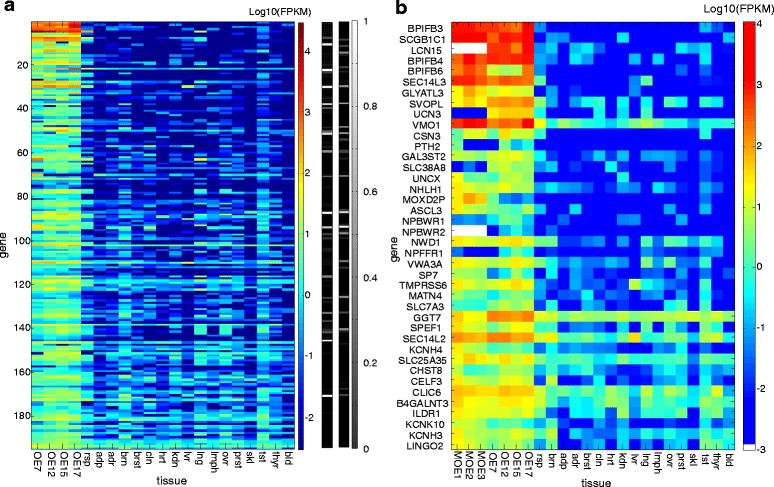
Genes with overexpression in human olfactory epithelium. **a** Expression profile of all 195 overexpressed genes in different human tissues (four olfactory epithelial and 17 controls). Right-hand side two columns show functional evidences in gray scale for every gene, based on GOSdb scores (right) calculated without the DS6-DS9 sources [10] and on PubMed searches (left). **b** Expression profile for a subset of the genes in (**a**), which are specifically mentioned in the Results section. White color - no mouse ortholog. Tissue abbreviations as in Fig 1, with the addition of : MOE; mouse olfactory epithelium., adp; adipose, adr; adrenal, brst; breast, cln; colon, hrt; heart, lmph; lymph, ovr; ovary, prst; prostate, skl; skeletal muscle, tst; testis, thyr; thyroid, bld; blood

We next identified a set of 196 non-olfactory receptor genes that were over- expressed (>X6) in human olfactory epithelium relative to the illumina BodyMap control tissues but not over-expressed in respiratory epithelium relative to the same controls (Fig. 2a and Additional file 3: Table S3). Using database and literature searches we were able to discriminate between genes with previous evidence of an olfactory role (36 genes, class A) and genes with little or no previous knowledge on such functional involvement (158 genes, class B). Class A genes include the expected well-known olfactory signal transduction genes such as *OMP*, *CNGA2*, *CNGA4, GNG13, ANO2, RTP1,* and *RTP2.* Additional genes in class A include biotransformation enzymes such as *UGT2A2*, and more (Additional file 3: Table S4). Notably, all class A genes are over expressed in mouse olfactory epithelium, except *NOS2*.

### New olfactory expression vistas

We subsequently examined the 158 class B genes and identified 109 genes that were overexpressed in human as well as mouse olfactory epithelium, highlighting their putative new olfactory role (Additional file 3: Table S5). These olfactory epithelium-enriched genes are described below, grouped by functional subgroups (Fig. 2b).

#### Non-olfactory GPCRs

We identified three neuropeptide receptors, *NPBWR1*, *NPBWR2* and *NPFFR*1 (Additional file 1: Figure S2). *NPBWR1* and *NPBWR2* have a role in regulating feeding behavior, energy homeostasis, neuroendocrine function, and modulating inflammatory pain [26] as well as in the regulation of emotion-related responses that affect autonomic functions [27]. *NPFFR*1 has a role in GnRH signaling of the reproductive axis [28] NPBWR1 and *NPBWR2* are receptors of the *NPB* and *NPW* neuropeptides. The peptide *NPB*, but not *NPW* shows sufficient expression (1.6 FPKM) and overexpression (X2.77) in human olfactory epithelium, to warrant notice.

#### Neuropeptides

This subgroup includes four neuropeptides: *UCN3* and *PTH2* (class B genes), and two more well-known (class A genes, *AVP*, *TAC4*) (Additional file 1: Figure S3). Two of these neuropeptides, *AVP* and *UCN3*, were respectively linked to the processing of social stimuli in sensory regions of the brain [29] and to social discrimination abilities via corticotropin-releasing hormone receptor type 2, being localized in nuclei functionally connected to the accessory olfactory system [30]. We note that *PTH2* is highly overexpressed in isolated olfactory sensory neurons (Additional file 3: Table S3 and Table S5), suggesting a yet undefined role in these cells.

#### Ion channels

Three voltage gated potassium channels (*KCNK10, KCNH3* and *KCNH4*) and a chloride channel (*CLIC6)* appear among class B genes (Additional file 1: Figure S4). KCNH channels are voltage-gated potassium channels with roles in cardiac repolarization, cellular proliferation and tumor growth [31]. All three genes show strong overexpression in isolated olfactory sensory neurons (Additional file 3: Table S3 and Table S5). Future scrutiny could uncover a possible role in olfactory epithelial differentiation or neurogenesis, as suggested [32]. *CLIC6* (X16.5 overexpressed) is a member of the chloride intracellular channel family, which functions as monomeric soluble proteins and as integral membrane chloride ion channels. In the soluble form they adopt a glutathione S-transferase (GST) fold, with an enzymatic activity [33]. In line with this, our data are consistent with function in olfactory epithelial cells other than the sensory neurons (Additional file 3: Table S3 and Table S5).

#### Solute carriers

Four proteins of this group are identified: *SVOPL*, member of the SCL22 family, *SLC25A35*, *SLC38A8* and *SLC7A3* (Additional file 1: Figure S5)*.* SVOPL is a putative synaptic vesicle glycoprotein and its affiliation with the SLC22 family suggests a role as organic ion transporters. Enhanced expression of SVOPL in the olfactory bulb and cerebral cortex has been reported [34], consistent with an olfactory role. Yet, based on the analysis of isolated olfactory sensory neurons (Additional file 3: Table S3 and Table S5), these genes may have a role in non-sensory cells of the olfactory epithelium. *SLC38A8* functions as a neuronal transporter with a broad amino acid transport profile and was suggested to have a key role in the glutamine/glutamate (GABA) brain cycle [35].

#### Transcription factors

Thirteen transcription factors, with little or no known olfactory involvement are overexpressed (Additional file 1: Figure S6). Of these we note the gene *ASCL3,* member of the achaete-scute complex (ASC) family which has a role in cell fate determination and differentiation of numerous tissues, including neuronal tissues [36]. In drosophila achaete-scute gene complex (AS-C) is a key component in developing of the macrochaetes sensory organs [37]. The paralog *ASCL1* is required for early development of olfactory neuron [38]. Three additional transcription factors are *UNCX*, suggested to participate in the regulation of neural progenitor cells proliferation and neuronal survival in the olfactory epithelium [39], and *SP7* which plays a role in the olfactory glomerular layer [5] and *NHLH1*, a neurogenesis transcription factor whose expression in mouse olfactory epithelium and vomeronasal organ during development has been reported [40]. The expression data provide further support for such roles. A specific mature olfactory sensory neuronal function is suggested only for one of the above, *UNCX* (Additional file 3: Table S3 and Table S5).

#### Other genes

Other genes for which we suggest a novel olfactory role include: 1) matrilin 4 (*MATN4*), a member of the von Willebrand factor A domain-containing protein family, involved in the formation of filamentous networks in extracellular matrix. In zebrafish *MATN4* expression was significantly increased following exposure of olfactory epithelium to the odorant phenylethyl alcohol in a potential context of memory formation [41]; 2) Leucine Rich Repeat and Ig Domain Containing 2 (*LINGO2*), reported to be expressed during mouse embryogenesis in a population of cells lying adjacent to the epithelial lining of the olfactory pit [42], and to be involved in the development of the olfactory pathway of mouse and zebrafish embryos [42]. 3) *SPEF1*, a microtubule-associated protein, that plays a role in the structural integrity of auditory sensory epithelium [43]; 4) *VMO1* a protein of the outer layer of the vitelline membrane of eggs which has an essential function in the antimicrobial barrier in avian eggs [44] and was suggested to function in tear integrity [45, 46]; 5) *EFR3B,* a palmitoylated plasma membrane protein, which is responsible for maintaining an active pool of the *PI4KA* enzyme at the plasma membrane. *EFR3B* was recently shown to function also as a direct regulator of GPCRs [47]. The high overexpression of three of the above genes, *LINGO2*, *SPEF1* and *EFR3B* in isolated sensory neurons is noteworthy (Additional file 3: Table S3 and Table S5).

Other than that we identified several RNA genes, linc RNA genes and antisense genes which might have a role in the regulation of the olfactory system, three transmembrane protein, two Squalene Transfer Proteins (*SEC14L2* and *SEC14L3*) and 24 other secreted proteins. Among the secreted proteins especially high over expression is observed for *SCGB1C1.*

### Odorant binding proteins (OBPs)

Our data indicate a complex situation with respect to olfactory epithelial overexpressed genes that might harbor odorant binding protein (OBP) function. In mouse 4 paralogs genes, Obp1a, Obp1b, Obp2a and Obp2b are highly overexpressed (Additional file 4: Table S6). These are “classical” OBPs, as indicated by their symbols, and indeed all four are highly expressed in the mouse sensory organ. However, in human only two of the four appear to have orthologs bearing identical symbols (OBP2A, OBP2B, Additional file 1: Figure S6). Surprisingly, these two genes do not show any enhanced expression in the human sensory tissue (under-expression of 0.1X and 0.32X respectively and a very low absolute values of 0.12 and 0.08 FPKM respectively). In fact OBP2A, OBP2B are overexpressed in testis and ovary (Additional file 1: Figure S8). This may indicate a case of functional misidentification.

The finding that human OBPs are likely to have no olfactory role is further corroborated by the recent OBP2B crystal structure, showing a structural features different from those of other mammalian OBPs, including a potentially reactive cysteine side chain within the binding pocket, which is most similar to human tear lipocalin [48].

**Fig. 3:**
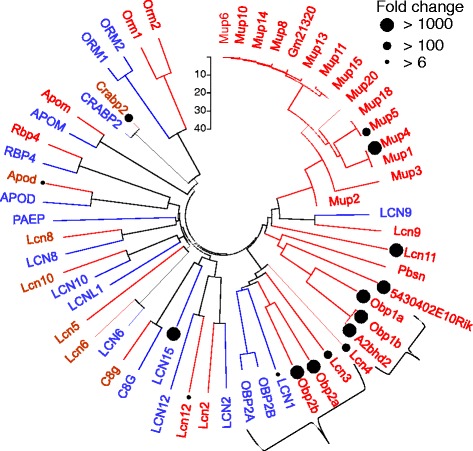
Phylogenetic analysis of the lipocalin family. The evolutionary branches of OBP1 and OBP2 are marked by curly brackets. Black circles indicate the fold change of a gene in olfactory epithelium relative to controls. The tree was constructed with MEGA6 [88] using the NJ method [87]. Red- mouse; blue - human

Alternative human functional OBPs may be gleaned in the broad scope orthology dendrogram of Fig. 3, showing sequence relationships as well as overexpression traits. A second gene sub-family - lipocalins with explicit symbol prefix LCN - appears to be relevant to human olfaction. In human there are 9 LCN genes while in mouse there are 10 such genes. In human, only two enhanced sensory organ expression: LCN1 and LCN15, the latter showing especially high overexpression. In contrast, a quite different subgroup of LCN genes shows mouse sensory prominence: Lcn3, Lcn4, Lcn10, Lcn11, with the latter being the strongest.

In addition, several BPI fold containing proteins which were previously suggested to function as odorant binding proteins [49] are extremely overexpressed (X10,000 or more) in human olfactory epithelium. Such striking result lends credence to a possible olfactory role, possibly in odorant carrying or removal. The null expression in the sensory neurons (Additional file 3: Table S5) is consistent with expression in non-neuronal secretory cells of the olfactory epithelium or sub-epithelium.

### Biotransformation enzymes

Biotransformation enzymes are involved in xenobiotic modification and clearance [50, 51]. Some of these enzymes have previously been reported to be expressed in the olfactory epithelium and suggested to play a role in odorant modification [52–54]. These include two cytochrome P450s (CYP2G1, CYP2A13) and one UDP glucuronosyl transferase (UGT2A2), which are indeed overexpressed in olfactory epithelium (Additional file 1: Figure S9, Additional file 3: Table S5). The mouse ortholog of CYP2G1, *Cyp2g1,* is a known olfactory-enriched protein [53, 55, 56] suggested to be involved in clearance of odorous compounds [52]. Another seven biotransformation enzymes show high (>X10) olfactory over expression (Additional file 1: Figure S9). Among these, for the aldehyde dehydrogenase ALDH3A1 and the glutathione peroxidase GPX6 there are previous indications for olfactory involvement [50, 57]. Four others, a glycine acyl transferase (GLYATL3), a galactose sulfotransferase (GAL3ST2), a gamma glutamyltransferase (GGT7) and a carbohydrate sulfotransferase (CHST8) have no reported olfactory role, and their specific olfactory epithelial expression is worthy of future scrutiny. Of note is that the only one among the abovementioned genes that has a strong enrichment in isolated sensory neurons is CHST8 (Additional file 3: Table S3 and Table S5).

Another biotransformation enzyme overexpressed in human olfactory epithelium is the dopamine beta-hydroxylase-like monooxygenase *MOXD2.* This enzyme harbors frequent loss-of-function mutations in some apes, toothed whales and baleen whales [58]. In human the gene inactivated due to a 2 exons deletion [59] that is fixated in the population, as indicated by 1000 genomes and DGV scrutiny. This pseudogenization is possibly connected to the impaired olfactory faculties in apes and monodontidae [58] as well as in human [60].

### Olfactory receptors

**Fig. 4:**
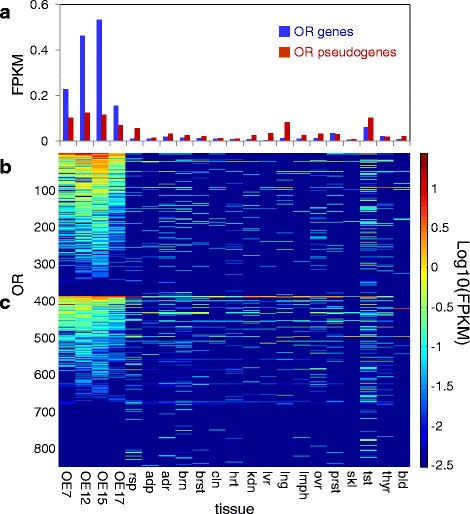
Expression profiles of OR genes. **a** the average expression of OR genes and pseudogenes per enumerated tissue. **b**, **c** respective expression profile for the same tissues for intact and pseudogenized genes. Tissue abbreviations as in Fig 2

We examined the expression profile of all mappable OR genes in human olfactory epithelium (Fig. 4, Additional file 5: Table S7). Using a cutoff of with FPKM > =0.01 [61] we observed that 88.6 % of the intact OR genes were expressed, while a much lower percentage (61.2 %) of the OR pseudogenes were expressed in at least one of the tissue samples. Further, intact OR genes had a significantly higher average expression level (0.35 ± 1.08 FPKM) as compared to OR pseudogenes (0.09 ± 0.33 FPKM, P = 3.7X10^−26^, Kolmogorov-Smirnoff test, Fig. 4). Moreover, we found a significant correlation between the predicted probability of the OR to encode a functional protein, computed by the CORP score [62] and its expression level (P = 3.62e-7, Kolmogorov-Smirnoff test, Additional file 1: Figure S10). Interestingly, in control non-olfactory tissues the relationship is inverted, whereby OR pseudogenes have a higher average expression than intact genes (Fig. 4). We note that among non-olfactory tissues, the highest expression of both OR genes and pseudogenes is in testis, confirming a previous report [61].

**Fig. 5:**
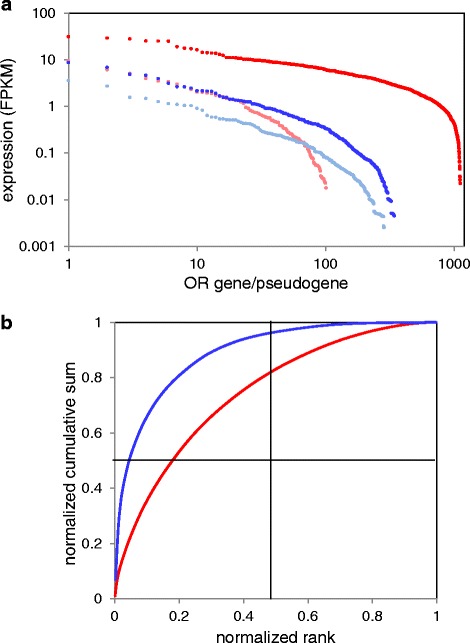
Rank plots for the expression values of human and mouse ORs. **a** Ranked expression values. Red and pink respectively represent intact and pseudogenized mouse OR genes; Blue and light blue respectively represent intact and pseudogenized human OR genes. **b** Normalized cumulative expression values, colors as in (**a**)

We asked about trends in expression levels across the OR repertoire. Our data clearly indicate that expression levels of different OR loci are highly heterogeneous. While for intact human OR genes the median is about 0.1 FPKM, the top OR is expressed at levels 100 fold higher, and the overall span is around three orders of magnitude (Fig. 5a). Human OR pseudogenes show a similar trend, suggesting that protein functionality may not be underlying correlate. It appears that variations in the effectiveness of transcription regulation may be at work. In mouse, while the overall expression is ~5-10 fold higher across the board (probably due to tissue purity differences), the inter-OR heterogeneity is less pronounced. Interestingly, the discrepancy in expression levels between intact and pseudogenized loci is much higher in mouse, possibly because of the recently of pseudogenization in a large majority human OR genes [60]. Of note is that both species show a skew in the inter-OR expression variation, and that this is much more pronounced in human. While in mouse ~18 % of the ORs contribute 50 % of to the cumulative OR expression, in human the value is ~5 % (Fig. 5b). This may be rephrased as indicating that in the sensory organ, the effective repertoire, in particularly in human, is only a small fraction of the nominal repertoire. Interestingly, the position of a given OR on the expression intensity scale portrays no ortholog-pair correlation (Additional file 1: Figure S11). It might be argued that the above observations could be misleading due to inaccurate calling of genes and pseudogenes in human. This is because of the fact that individual human genomes contain a high number of deleterious variations that turn intact genes into pseudogenes in some individuals (segregating pseudogenes) [10]. We therefore redrew Figs. 4, 5 without all 282 OR loci reported to harbor segregating pseudogenes. The assignment of loci to segregating pseudogene status was done base on our previous data [10] that integrated 13 different resources, including the 1000 genomes project as well as exome sequences of over 1000 individuals, including deleterious SNPs, indels and CNVs. This process thus captured all segregating pseudogenes with an allele frequency >0.001 in the human population. The probability that an individual that underwent transcriptome analysis has rare private deleterious OR mutations is further diminished based on our statistics [10] that a typical individual genome has on average only 46 affected OR loci out of the population’s 282. The results shown in Additional file 1: Figure S12 suggest a very small effect as compared to Fig. 4. Likewise, while the results relevant to Fig. 5a seen in Additional file 1: Figure S13A show somewhat different trends due to the different gene count, the normalized curves relevant to Fig. 5b (Additional file 1: Figure S13B) are nearly indistinguishable with and without omission of segregating pseudogenes.

Interestingly, within the mouse OR genes we find that class II ORs have significantly higher expression level that class I ORs (P = 7.2*1e-15, Chi Square), consistent with the recent findings of [63]. Class II ORs have a higher expression also within the human OR expression data, although the difference was not significant (0.06, Chi Square), perhaps due to the low count of human class I receptors.

### Inter-individual patterns of OR expression

**Fig. 6:**
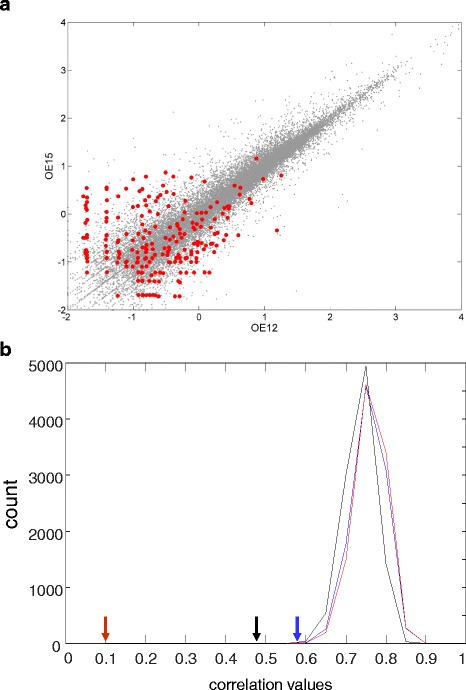
Inter-individual patterns of OR expression. **a** Inter-individual correlation of the ORs expression level (red) versus the whole genome (grey) for samples OE12 and OE15. Data are presented on a log10 scale of the FPKM values. The Pearson correlation values are 0.35 for the OR genes and 0.9 for the whole genome. The complete data set is shown in Additional file 1: Figure S12. **b** Pearson correlation values for OE pairs shown by arrows: OE12,OE15 - red, OE12, OE17 – black, OE15, OE17 - blue. Also shown are distributions of Pearson correlation values obtained from 10,000 random sets for the respective pairs with same color code. The respective P values for the above pairs are <0.0001, 0.0004 and <0.0001. For the other three pairwise comparisons involving OE7 the P values were 0.0801 (OE7,OE12), <0.0001 (OE7, OE15), 0.46 (OE7-OE17), i.e. not significant (see text)

The foregoing analyses were performed on values averaged across the four human samples employed. We asked whether there was inter-individual variations in the expression of OR genes. Such across-sample variations are indeed visible in Fig. 4. For further validation, we computed the six pairwise correlation values for the four human olfactory epithelial samples and indeed observed lower correlation values for OR genes as compared to the complete gene set (Fig. 6a, Additional file 1: Figure S14). However such results could stem from noise related to the fact that ORs typically have low expression values. To address this, we analyzed 10,000 random subsets of a similar size to that of the OR set which obey the same FPKM distribution as the OR genes. For OE12, OE15 and OE17 this simulation demonstrated that the correlation values for the OR gene set lie completely outside the distribution for the 10,000 control gene sets (Fig. 6b). Thus for these tissues the expression level of the ORs is significantly more variable than that of other genes. When OE7 was compared to each of the other three tissues, no significant inter-individual differences were observed (Fig. 6b legend). This is likely due to the 5 fold lower reads for this tissue.

A potential weakness of the conclusions regarding inter-individual differences in OR expression patterns is that some such difference could arise due to differences in the counts of intact OR alleles in different individuals [10]. To address this, we redrew Fig. 6 and Additional file 1: Figure S14 in a version that excludes all gene loci previously reported [10] to harbor segregating pseudogenes (Additional file 1: Figures S15-16). The results were nearly identical to the original.

### Genomic structure of OR transcripts

**Fig. 7:**
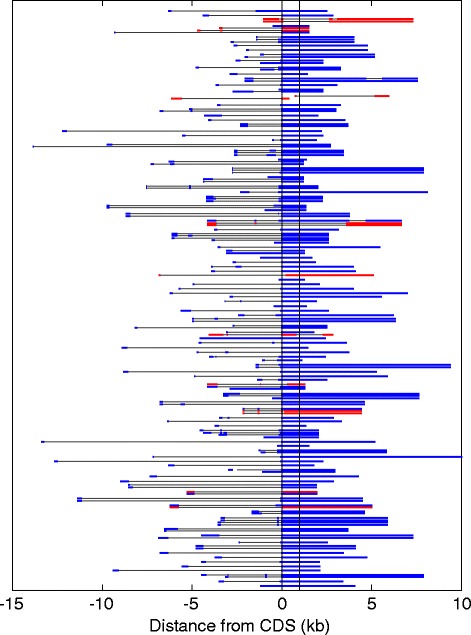
Genomic maps of OR transcripts. Transcripts are presented on a genomic scale, phased by the OR open reading frame (vertical thin lines). Thick blue/red lines are exons and thin gray lines are introns. Narrowly spaced transcripts are for the same OR gene. Transcripts with a disrupted open reading frame are in red. These are: OR52K1, OR2V1, OR6C75, OR4M1, OR51L1, OR2A1, OR10H4, OR2K2, OR2J3 and OR6F1

**Fig. 8:**
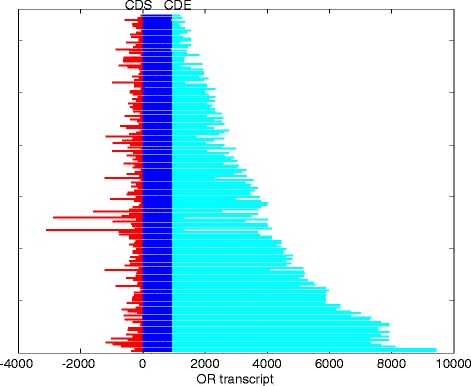
The OR transcripts. The transcripts are justified to the initiating methionine. The 5’ UTR region is in red, coding region in blue and 3’ UTR in light blue

We used uniquely-mapped RNAseq reads from all human olfactory epithelial samples to assemble transcripts for intact OR genes. After a curation process we obtained 311 transcripts representing 210 intact OR genes. Of these, 120 transcripts with expression level <1.0 FPKM were excluded to avoid inaccuracies in transcript assembly, and the remaining 163 transcripts (encoded in 100 genes) were further analyzed (Fig. 7 and Additional file 1: Figure S17, Additional file 6: Table S8). The OR transcripts obtained were 4097 ± 2053 bp long and spanned genomic lengths of 8103 ± 3464 bp. The coding exon contains an open reading frame of 940 ± 16 bp and 3’ UTR of 2777 ± 2047 bp (Fig. 8). The 5’ UTR is 389 bp long, and contains 0–3 non-coding exons (Fig. 7). The number of splice variants per gene varies from 1 to 5 (Fig. 7). Of note are 10 OR genes with a seemingly non-functional transcript that skips the initiating methionine (Fig. 7), suggesting a yet unreported OR pseudogenization mechanism. Eight of these aberrant transcripts show the co-existence of both functional and non-functional transcripts, indicating the presence of two different alleles.

Our data provide repertoire-wide information on the gene structure of intact human OR genes. For comparison, we screened the AceView gene model repository [64], calculated from public expressed sequences and found 279 AceView transcripts that harbor also untranslated regions. However, only 36 of them (12.9 %) are spliced, with a substantial shorter the 3’UTR (159 ± 352 bp), probably due to the insufficient coverage in non-olfactory cDNA libraries. In the case of one gene, *OR51E2,* known to be highly expressed in prostate [65], we identified potential disparate tissue-specific promoters. The proximal promoter appears to be active in olfactory epithelium, while the distal promoter – in prostate (Additional file 1: Figure S18). The latter is inferred from AceView data, as well as well as from the prostate Illumina Body map data.

### The OR promoter region

**Fig. 9:**
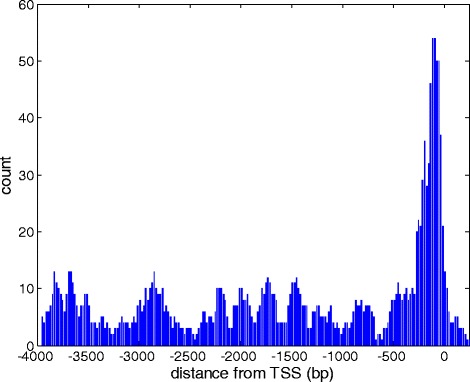
Upstream binding site profile for the EBF family of transcription factors. Shown are summed counts of predicted binding sites in a 4 kb interval upstream to the transcription start site (TSS). This applies only OR genes for which a transcript defines one or more TSS

We used the Genomatix RegionMiner tool to search for significant over representation of transcription factors binding sites (TFBS) immediately upstream from the inferred transcription start site. This was done jointly analyzing all 160 OR transcripts, with a comparison to the Genomatix genome-wide promoter region collection. The top 10 TFBS of all belonged to homeodomains class, including Genomatix families V$LHXF and V$HBOX. These respectively include the transcription factors LHX2 and EMX2, previously shown to be required to the expression of OR genes [66, 67]. The results are also in agreement with a similar analysis of mouse OR promoters [21, 68]. Repeating the analysis with a whole genome reference, or with promoters with matched low GC content (<60) to that of presumed OR promoters, identified an enrichment of the V$NOLF family of the Early B-Cell Factor (EBF) proteins, involved in the expression of ORs [39]. These V$NOLF TFBS are clustered in a distance of 100–300 bp from the transcription start site (Fig. 9), in broad agreement with the mouse data [21, 68]. The appearance of a distinct propensity peak for V$NOLF suggests that our OR transcription start site inference is adequate.

## Discussion

### Comprehensive transcriptome

The human olfactory epithelium is relatively difficult to obtain in a high quality, due to anatomical inaccessibility and heterogeneous boundaries. Specifically, there is variable contamination with non-sensory respiratory epithelium [6]. We succeeded in doing the analysis by examining biopsy and autopsy samples form 17 different human individuals, and selecting the ones most-enriched for known olfactory markers. Further, the gene-specific signals were filtered with respect to respiratory epithelial over-expression. This enables us to report a dependable and comprehensive transcriptomic view of this human sensory tissue.

### New vista of olfactory auxiliary genes

We identified a large number of olfactory auxiliary genes, those playing a role in transduction, development and maintenance [10]) (Additional file 3: Table S3 and Additional file 7: Table S9). Our working hypothesis was that many of these would be highly expressed in human olfactory epithelium. It is clear that such correlation may not be perfect, i.e. genes that play a role in development and maintenance are not necessarily olfactory specific and expressed at higher levels. Still, there is a relatively broad consensus that tissue-specific expression has functional implications [21, 69, 70].

It is also noteworthy that tissues are often heterogeneous, and a prominent example is olfactory mucosa, which includes sensory and supporting cells, basal progenitor cells and subepithelial Boman’s gland cells. Further, tissue contamination may occur, e.g. with respiratory epithelium. However, as we set our overexpression cutoff of X6, a 10 % contaminating tissue would have to show X60 overexpression of a gene, and such high spurious expression signal would be easily excluded by our use of respiratory epithelium control. To summarize, equating olfactory epithelial over-expression with chemosensory function should be taken with caution, but is a relatively high probability premise.

In a first group are genes that are already well-known to play a key role in olfactory function. Some such genes are found to be significantly overexpressed in the sensory tissue (e.g. *OMP*, *GNG13* and *ANO2*), while others were not, due to a broader appearance in non-olfactory tissues (e.g. *GNAL* and *ADCY3*).

In the second group are genes that have very few or no previous publications on olfactory involvement. Some of these genes are also highly expressed in mouse olfactory epithelium, as also reported [32]. Such inter-species concordance increases the probability of olfactory involvement for those genes.

### Apparent species-specific genes

Among the 194 olfactory over-expressed genes in human, we identified 58 that do not show over expression in mouse, or do not have a mouse ortholog. Such discordance suggests inter-species functional differences between mammals that need to be further elucidated. In an example, we showed the different patterns of expression of the broadly-defined lipocalin family members, where human and mouse show differential expression in disparate members of the family. Likewise, while in mouse several TAAR genes are overexpressed, in human only one paralog (TAAR5) shows such differential expression. These results may suggest that in evolution, different protein family members assume a chemosensory role. We note that some of these inter-species discrepancies could rest in the fact that the mouse data were obtained from samples pooled from four animals, which likely included a better representation of the olfactory epithelium, while the human anatomical sampling was more heterogeneous.

### Expression of olfactory receptors

The RNAseq data accumulated in this study provide an informative view on the expression of OR genes in olfactory epithelium and other tissues. In the olfactory tissue we observed the expression of nearly 90 % of the intact OR repertoire, but only ~60 % of all OR pseudogenes. Further, the expression distribution curve of pseudogenes is shifted to ~ X4 lower intensity relative to intact genes (Fig. 4a). This may be due to the accumulation of promoter mutations or to nonsense mediated decay. In non-olfactory (ectopic) tissues the typical expression of intact OR goes down about X7, while at least in some such tissues the pseudogene expression is not much altered. This may reflect in part the functional feedback mechanism that selects against pseudogene expression [71].

An intriguing finding is the three orders of magnitude difference in expression intensity of different members of the OR repertoire, both in human and in mouse. This phenomenon has been previously observed in mouse by other methodologies [20, 72], and may be accounted for in part by variations in transcription regulatory efficiency along OR gene clusters. Here we add a functionally relevant dimension to this observant, namely that the decline of OR expression in the rank curve is much faster in human as compared to mouse, hence the overall (integral) repertoire expression is considerable weaker in human (Fig. 5b).

Finally, we observe significant inter-individual differences in the across-repertoire pattern of OR expression as compared to whole genome measures, consistent with previous reports [73, 74]. It important to ask whether this phenomenon is a true inter-individual difference, with a potential genetic basis, or due to sampling artefact. The expression of different ORs is known to vary strongly among different anatomical zones of rodent olfactory epithelium. Thus, one possible explanation could be that in different individuals tissue samples were from different functional zones, though zonal OR expression has not yet reported in human. If the mechanism is genetic after all, one potential explanation is inter-individual difference in OR regulatory sequences. Alternatively, genetic variations elsewhere could be responsible. Clarifying these issues requires to perform extensive expression quatitativew trait loci (eQTL) experiments, whereby genome sequencing a transcriptome analyses is concomitantly performed on the same individuals. As this far transcends the scope of the present study, it should be clearly stated that the inter-individual expression variation trends reported here should be taken with significant caution. Likewise, if one wishes to extend such results to mouse, this would necessitate comparing several different mouse trains, again beyond the present scope.

### The OR transcript

Despite the low expression of individual OR genes, we succeeded in defining the exon-intron structure of 160 transcripts in 100 OR genes, those that show higher expression. We are aware of the possibility that because of the relatively low coverage, some of the features described herein represent transcript-mapping artifacts. The 160 transcripts elucidated may well be a representative sample, as a correlation between gene structure deviations and expression strength is not very likely, and as no OR family bias has been observed along the expression rank curve (not shown).

The OR transcripts have certain common features. These include 1–4 short 5’ non-coding exons with considerable alternative splicing: 43 of the 100 genes having 2–4 splice variants; long last 3’ exon which contains the entire OR coding region, i.e. no introns are seen within the coding region. At the same time, considerable structural variability is seen in 5’ exon count, as well as intron length and 3’UTR length (Fig. 7). One interesting case is that of *OR51E2*, which in olfactory epithelium contains the coding exon spliced to one 5’ non-coding exon. In prostate, the same coding exon is spliced to a remote 5’ non coding exon of a neighboring OR pseudogene *OR51C1P*. This chimeric transcript is known to be highly expressed in prostate, and appears to bear a functional role in this tissue [75].

OR genes have undergone a massive recent evolutionary process of pseudogenization [60]. OR pseudogenes do not arise by retroposition, but rather by gradual accumulation of point mutations and indels that render the encoded protein aberrant. We report here an additional pseudogenization process that affects 15 transcripts in 10 OR genes with an intact open reading frame. These events stem from inappropriate slicing, eliminating part or the entire coding region. We note that only in three of the cases no intact OR-encoding splice variant remains.

The analysis performed allowed us to accurately define for the first time the transcription start site (TSS) of 100 human OR genes. We subsequently analyzed the transcription regulatory elements immediately upstream to the TSS. The results indicated an enrichment of transcription factor binding site signature belonging to the large homeobox family in that region. In addition, we found a consensus localization of a specific transcription factor binding site subfamily (Olf/EBF). In mouse, these binding sites are known to be present in promoter regions of the OR repertoire, and to play a key role in OR transcription [21, 68].

## Conclusions

We report the first RNAseq study of human olfactory epithelium, aimed at obtaining a whole transcriptome overview of the sensory tissue. Our work reveals nearly 200 olfactory-enriched non-receptor transcripts, 80 % of which have not yet been implicated in chemosensory function, thus providing a platform for future discovery.

Our study further allowed us to quantitate the expression levels of most (90 %) of the ~400 intact human olfactory receptor (OR) genes. The expression levels of different paralogous members of the OR repertoire span a 1000-fold range, suggesting a strong imbalance among different odorant specificities. Further, we obtained convincing hints that different human beings show different cross-repertoire expression patterns. Having successfully assembled transcripts for 100 OR genes, we observed prevalent genome-encoded mutations that render some transcripts inactive despite the fact that the protein coding region is intact, an unreported mechanism for OR pseudogenization.

In sum, our transcriptome study offers important observations on genes that underlie olfactory function and provides a basis for significant future work.

## Methods

### Samples collection

#### Human olfactory epithelium samples

We obtained 14 biopsy tissues and three autopsy tissues of human olfactory epithelium biopsies dissected from the superior ethmoturbinal, the cribriform plate and the superior septum [73, 76] during elective surgeries of the samples donors. We used qPCR (see below) to characterize the olfactory content of 12 of the biopsies samples (Additional file 1: Figure S11A), and measure the expression level of olfactory markers (CNGA2, GNAL, OMP) versus respiratory markers (KRT13 [77] and TMPRSS11D [78]). Three samples (OE12, OE15 and OE17) were selected for RNAseq and analysis. A third biopsy sample (OE7) was selected for analysis based on the expression of the olfactory markers in the RNAseq results (Additional file 1: Figure S1B and Table S1). The other samples were excluded from the analysis, as they failed to show expression of olfactory markers.

The study was approved by the Ottawa Health Science Network Research Ethics Board and the Ethics Board of University Rehabilitation Institute (Ljubljana, Slovenia). All participants provided informed consent.

#### Mouse samples

C57BL/6 mouse olfactory epithelium and mouse olfactory bulb RNA (Additional file 1: Table S1 samples MOE1 and MOB) were collected in pool from four adult female Trpm5-GFP mice crossed with C57BL/6 for over 10 generations. These experiments were performed according to protocols approved by the University of Colorado Anschutz Medical Campus Institutional Animal Care and Use Committee. BALB/c olfactory epithelium RNAseq data were kindly provided by Wen-Hsiung Li, Academia Sinica, Taiwan [79]. Of note, the MOB sample was used only for presentation in Additional file 1: Figures S2-S6, S8, and S9.

#### Mouse olfactory sensory neurons

Single dissociated cells of olfactory sensory neurons were randomly selected under a light microscope and seeded into a tube containing cell-lysis buffer by mouth pipetting. After reverse transcription and global amplification of cDNA, diagnostic PCR for four mature olfactory sensory neuron marker genes (olfactory marker protein (*Omp*); guanine nucleotide binding protein; alpha stimulating, olfactory type (*Gnal*); cyclic nucleotide gated channel alpha 2 (*Cnga2*) and adenylate cyclase 3 (*Adcy3*)) was carried out using the diluted amplified cDNA. Samples with the presence of all of four markers were selected for library preparation and sequencing by following the Illumina protocol. After aligning the read data to the mouse genome (mm9) an expression profile was generated using only the uniquely mapped reads and those that are mapped to exons. The number of reads per gene was further divided by the total number of the sample mapped reads (reads per million, RPM). The data presented in this manuscript is the average of nine olfactory sensory neurons collected from female Mus musculus domesticus (C57BL/6, B6) and an hybrid mice from cross of male Mus spretus (Spretus) and female B6.

#### Control samples

The Illumina human body map 2.0 project data containing RNAseq of sixteen normal human tissues were used in this study as controls. Data were obtained directly from the company.

### Experimental procedures

#### Reverse transcription reaction

cDNA was synthesized with the SuperScript® First-strand Synthesis System for RT-PCR (Invitrogen, Carlsband, CA, USA) according to the manufacturer’s instructions.

#### Quantitative real-time polymerase Chain Reaction (qPCR)

qPCR reactions were performed using TaqMan® Gene Expression Assays (Applied Biosystems, Foster City, CA, USA). A total of 2ul of cDNA was added with 2.5ul of water and 0.5ul TaqMan® Gene Expression Assay to 5 ul of TaqMan® universal PCR Master Mix (Applied Biosystems) and the resulting 10 ul reaction mixtures were loaded onto a 96-well PCR plate. We used eight different TaqMan® Gene Expression Assays including three housekeeping genes with the following assays IDs: Hs01087269_s1 (OMP), Hs00181836_m1 (GNAL), Hs00864448_s1 (RTP1), Hs01377537_m1 (CNGA2), Hs00975370_m1 (TMPRSS11D), and ACTB (Hs99999903_m1) and GAPDH (Hs99999905_m1) as housekeeping genes.

Next generation sequencing-OE, MOE1 and the olfactory respiratory sample were sequenced at the biological services of the Weizmann institute using the Illumina Genome Analyzer platform (Illumina GA IIx) and the standard Illumina protocol at the experiment time. Samples OE12-OE17 were sequenced at Macrogene (Seoul, Korea). RNA samples were evaluated by ultraviolet spectroscopy for purity and concentration (NanoDrop, Wilmington, DE) and were assessed further for RNA integrity on the Agilent Bioanalyzer (Santa Clara, CA). Libraries were prepared using the Illumina mRNA-seq Sample Preparation Kit (San Diego, CA) and validated with an Agilent Bioanalyzer (Santa Clara, CA).

### Data analysis

#### Gene expression quantification

Reads of human olfactory epithelium were aligned to the hg19 reference genome using Tophat 2.0.4 [80]. Quantification of expression level was performed with Cufflinks v2.1.1. [81] using Ensembl transcripts and HORDE build#43 annotation [10] of OR genes and pseudogenes. To enable downstream calculations, 0 FPKM values were set to 0.003. Mouse data were analyzed with the same procedures using Mm10 as a reference genome. The GeneAlaCart tool of the GeneCards database [82] was used to find the mouse ortholog of each human gene.

#### Differential expression

To test the statistical significance of over expression in the olfactory epithelium versus the controls we used HTSeq [83]] to count the number of uniquely mapped reads per gene, following by the R package DESeq [84], using the illumina Body Map samples as control tissues. Genes with a fold change ≥ 6 and p <0.001 were considered as statistical significant. To overcome contamination from respiratory epithelium genes with expression in the respiratory epithelium that exceeded 0.1 of the expression in the olfactory epithelium were removed. We also included genes with a significant over expression in mouse olfactory epithelium provided that their over expression in human olfactory epithelium was above 6. The same analysis was applied to the mouse olfactory epithelium data. In the absence of mouse controls at the time that the analysis was performed, we used the illumina Body Map samples as controls [10]. Later on, with the progress of the mouse ENCODE project, we verified the over expression of the significant genes against a set of 11 mouse tissues (bone marrow, cerebellum, cortex, heart, intestine, kidney, liver, lung, spleen, testes, thymus) from the Mouse ENCODE project (http://chromosome.sdsc.edu/mouse/download.html).

#### Prior information on overexpressed genes

To associate between a given gene and olfaction we performed an automatic search in PubMed with the gene symbols of the olfactory enriched genes and a set of olfactory related keywords (olfactory;olfaction;odorant;odor;chemosensory;smell). Followed by a manual curation of some genes, the number of PMID hits was used to classify genes into class A or B.

#### Analysis of the lipocalin protein family

Most of lipocalin family members have no orthologs. Therefore over expression of mouse lipocalin genes were calculated using Mouse ENCODE data (http://chromosome.sdsc.edu/mouse/download.html) while over expression of human lipocalins was calculated relative to the human Illumina Body Map. Only tissues that are shared between the two control data sets were used in the calculation (adipose, brain, heart, kidney, liver, lung and testis), although the fold change of the over expressed genes did not altered when including the complete data set.

#### Assembly of OR isoforms

Uniquely mapped reads of all four studied olfactory epithelium samples were joined to assemble the OR transcripts. We used all reads from the OR territories defined as HORDE OR clusters [10] extended by 300 kb to the 5’ and 3’ of each cluster as an input to cufflinks, and applied the parameters -A 0.15 --trim-3-dropoff-frac 0.15. Cuffcompare [81] was used for gene identification. Transcripts encoded on the opposite strand of the OR genes and transcripts that are suspected as polymerase run-on fragment (cuffcompare classcodes x, s and p) were removed. We also removed isoforms with expression level <0.15 % of the major isoform of a gene. Transcripts with a single exon of > 5 kb length were curated by manual inspection.

#### Testing inter-individual differences of

To test the significance of OR expression we used all 264 OR genes that are expressed in all olfactory epithelium tissues and compared their expression to a control set of 15,846 protein coding genes which are also expressed in all tissues. The distribution of the ORs was calculated. We then randomly selected 264 control genes such that their distribution will follow that of the ORs tested by Kolmogorov-Smirnov *p* > 0.05. The process was repeated 10,000 times. Samples OE12-17, which proved to contain the highest olfactory epithelium content, and show the highest similarity, were used for this test.

#### Data-mining of AceView gene model

AceView transcripts [64] which overlap the OR coding regions were downloaded using the UCSC TableBrowser tool of the [85]. We then applied cuffcompare software against a gtf file of HORDE genes to assign the transcripts to the OR genes. Transcripts that overlap the opposite strand (cuffcompare class code x) were removed.

#### Promoter analysis

The tool RegionMiner of Genomatix was used to search for over representation of TFBS in the OR upstream regions. We used an interval of −750 bp and +250 of the TSS of each transcript. Transcripts representing the same OR gene were included if their upstream regions did not overlap by ≥ 500 bp. The analysis compared the sequences to a library of RefSeq promoters.

## Abbreviations

OR, olfactory receptor; TFBS, transcription factor binding site; TSS, transcription start site; UTR, untranslated region

## Additional files

Additional file 1: Table S1, Figures S1-S14.(PPTX 2640 kb) Additional file 2: Table S2. Whole genome expression of the studied samples (in FPKM). FC_OE, the fold change of the human olfactory epithelium relative to the controls; FC_resp, the fold change of the human respiratory epithelium relative to the controls; FC_MOE, the fold change of mouse olfactory epithelium relative to the controls, FC_MOB, the fold change of mouse olfactory bulb relative to the controls. NoOrth- no mouse ortholog. OSN- olfactory sensory neurons in RPM (reads per million). (XLSX 22311 kb) Additional file 3: Table S3. Olfactory over expressed genes. Tissue abbreviation: HOE, human olfactory epithelium; MOE: mouse olfactory epithelium; MOB: mouse olfactory bulb; RES: respiratory epithelium. Numbers in these columns are the averaged fold change relative to the illumina body map control samples (CTRL; FPKM), MOE_encode: the average fold change of mouse olfactory epithelium relative to mouse ENCODE data. OSN- olfactory sensory neurons. #pub- number of associated publications that were found in the automatic literature search. **Table S4.** Olfactory over expressed genes with prior evidences of an olfactory role. HOE: the fold change of human olfactory epithelium relative to the control tissues. MOE: the fold change of mouse olfactory epithelium relative to the control tissues. The 16 illumina Body Map tissues were used as controls in the calculation. MOE_encode: the fold change of mouse olfactory epithelium relative to 11 mouse ENCODE tissues. OSN- the fold change in mouse olfactory neurons relative to mouse olfactory epithelium. In bold- genes mentioned in the manuscript text. Gene symbols are from HGNC (http://www.genenames.org/). **Table S5.** Olfactory over expressed genes with little or no previous knowledge on olfactory role. See Additional file 3: Table S4 for more details. Symbols with * are from GeneCards (http://www.genecards.org/), with an informal gene name. (XLSX 1879 kb) Additional file 4: Table S6. Expression of the lipocalin family members. Mouse was calculated relative to Mouse ENCODE data (http://chromosome.sdsc.edu/mouse/download.html), see methods. (XLSX 14 kb) Additional file 5: Table S7. Expression profile of the OR repertoire. (XLSX 161 kb) Additional file 6: Table S8. Genomic coordinates of the OR transcripts (in gtf format, hg19). (GTF 70 kb) Additional file 7: Table S9. Genes that are discussed in the text. (XLSX 37 kb)
