# Supplementary material for: The human olfactory transcriptome
Source: BMC Genomics. 2016 Aug 11;17:619. doi: 10.1186/s12864-016-2960-3 (PMC4982115; doi:10.1186/s12864-016-2960-3)
Supplement: Additional file 1: — Table S1, Figures S1-S14.(PPTX 2640 kb) [file 12864_2016_2960_MOESM1_ESM.pptx]

## Slide 1
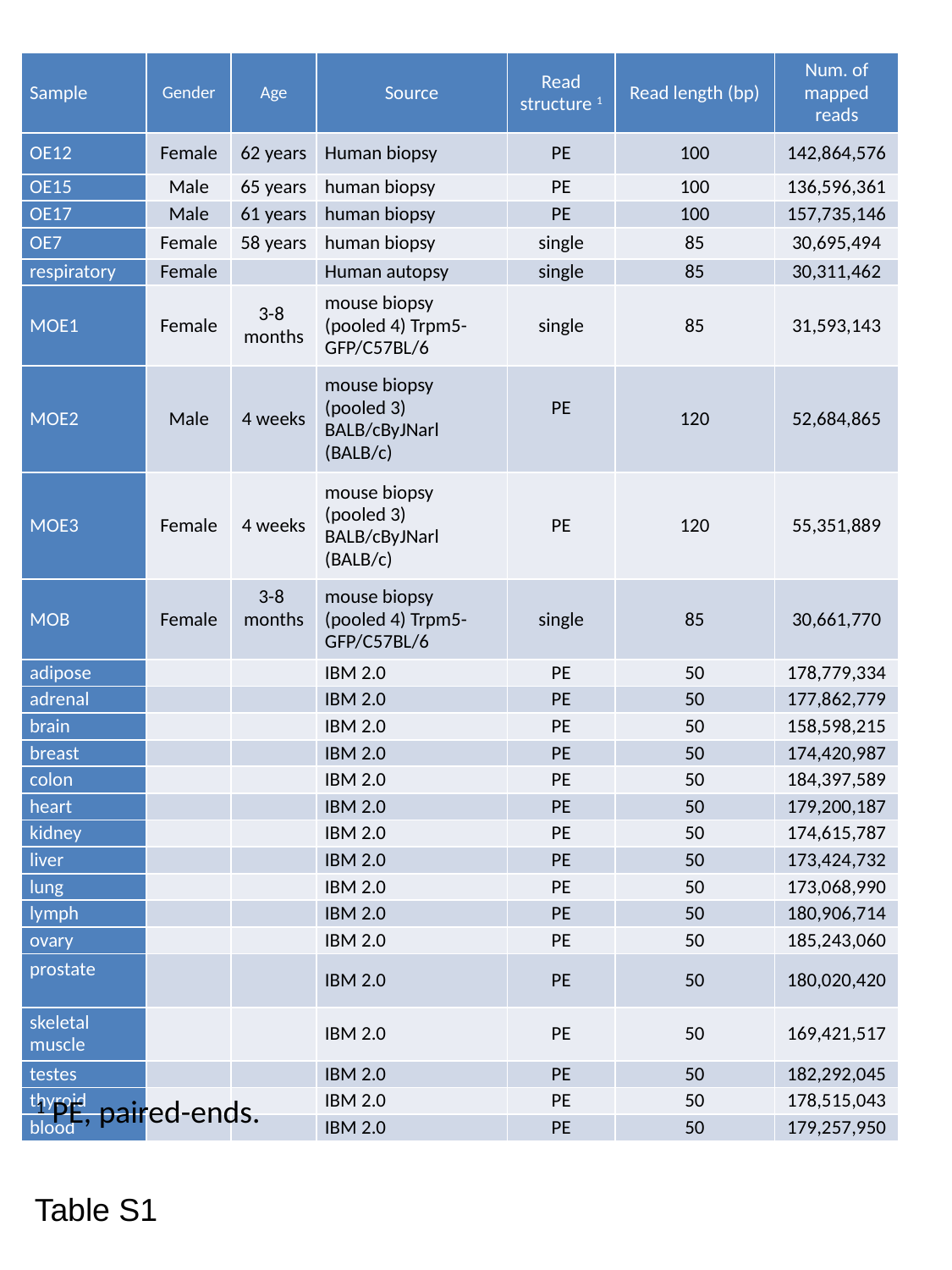

| Sample | Gender | Age | Source | Read structure 1 | Read length (bp) | Num. of mapped reads |
| --- | --- | --- | --- | --- | --- | --- |
| OE12 | Female | 62 years | Human biopsy | PE | 100 | 142,864,576 |
| OE15 | Male | 65 years | human biopsy | PE | 100 | 136,596,361 |
| OE17 | Male | 61 years | human biopsy | PE | 100 | 157,735,146 |
| OE7 | Female | 58 years | human biopsy | single | 85 | 30,695,494 |
| respiratory | Female | | Human autopsy | single | 85 | 30,311,462 |
| MOE1 | Female | 3-8 months | mouse biopsy (pooled 4) Trpm5-GFP/C57BL/6 | single | 85 | 31,593,143 |
| MOE2 | Male | 4 weeks | mouse biopsy (pooled 3) BALB/cByJNarl (BALB/c) | PE | 120 | 52,684,865 |
| MOE3 | Female | 4 weeks | mouse biopsy (pooled 3) BALB/cByJNarl (BALB/c) | PE | 120 | 55,351,889 |
| MOB | Female | 3-8 months | mouse biopsy (pooled 4) Trpm5-GFP/C57BL/6 | single | 85 | 30,661,770 |
| adipose | | | IBM 2.0 | PE | 50 | 178,779,334 |
| adrenal | | | IBM 2.0 | PE | 50 | 177,862,779 |
| brain | | | IBM 2.0 | PE | 50 | 158,598,215 |
| breast | | | IBM 2.0 | PE | 50 | 174,420,987 |
| colon | | | IBM 2.0 | PE | 50 | 184,397,589 |
| heart | | | IBM 2.0 | PE | 50 | 179,200,187 |
| kidney | | | IBM 2.0 | PE | 50 | 174,615,787 |
| liver | | | IBM 2.0 | PE | 50 | 173,424,732 |
| lung | | | IBM 2.0 | PE | 50 | 173,068,990 |
| lymph | | | IBM 2.0 | PE | 50 | 180,906,714 |
| ovary | | | IBM 2.0 | PE | 50 | 185,243,060 |
| prostate | | | IBM 2.0 | PE | 50 | 180,020,420 |
| skeletal muscle | | | IBM 2.0 | PE | 50 | 169,421,517 |
| testes | | | IBM 2.0 | PE | 50 | 182,292,045 |
| thyroid | | | IBM 2.0 | PE | 50 | 178,515,043 |
| blood | | | IBM 2.0 | PE | 50 | 179,257,950 |
1 PE, paired-ends.
Table S1

## Slide 2
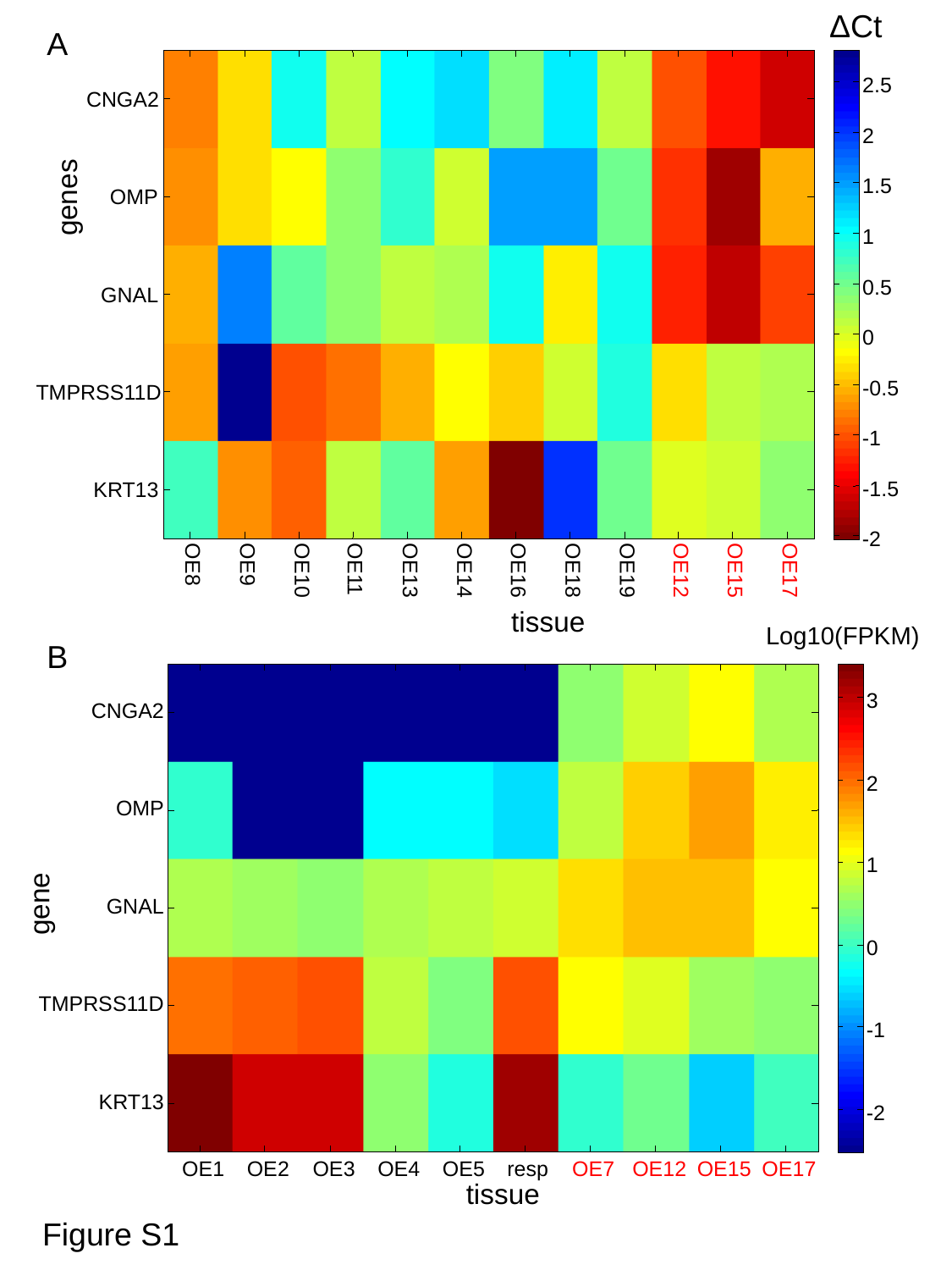

ΔCt
2.5
CNGA2
OMP
GNAL
TMPRSS11D
KRT13
2
1.5
1
0.5
0
-0.5
-1
-1.5
-2
OE8
OE9
OE11
OE10
OE13
OE14
OE16
OE18
OE19
OE12
OE15
OE17
genes
tissue
A
Log10(FPKM)
3
CNGA2
OMP
GNAL
TMPRSS11D
KRT13
2
1
gene
0
-1
-2
OE1
OE2
OE3
OE4
OE5
resp
OE7
OE12
OE15
OE17
tissue
B
Figure S1

## Slide 3
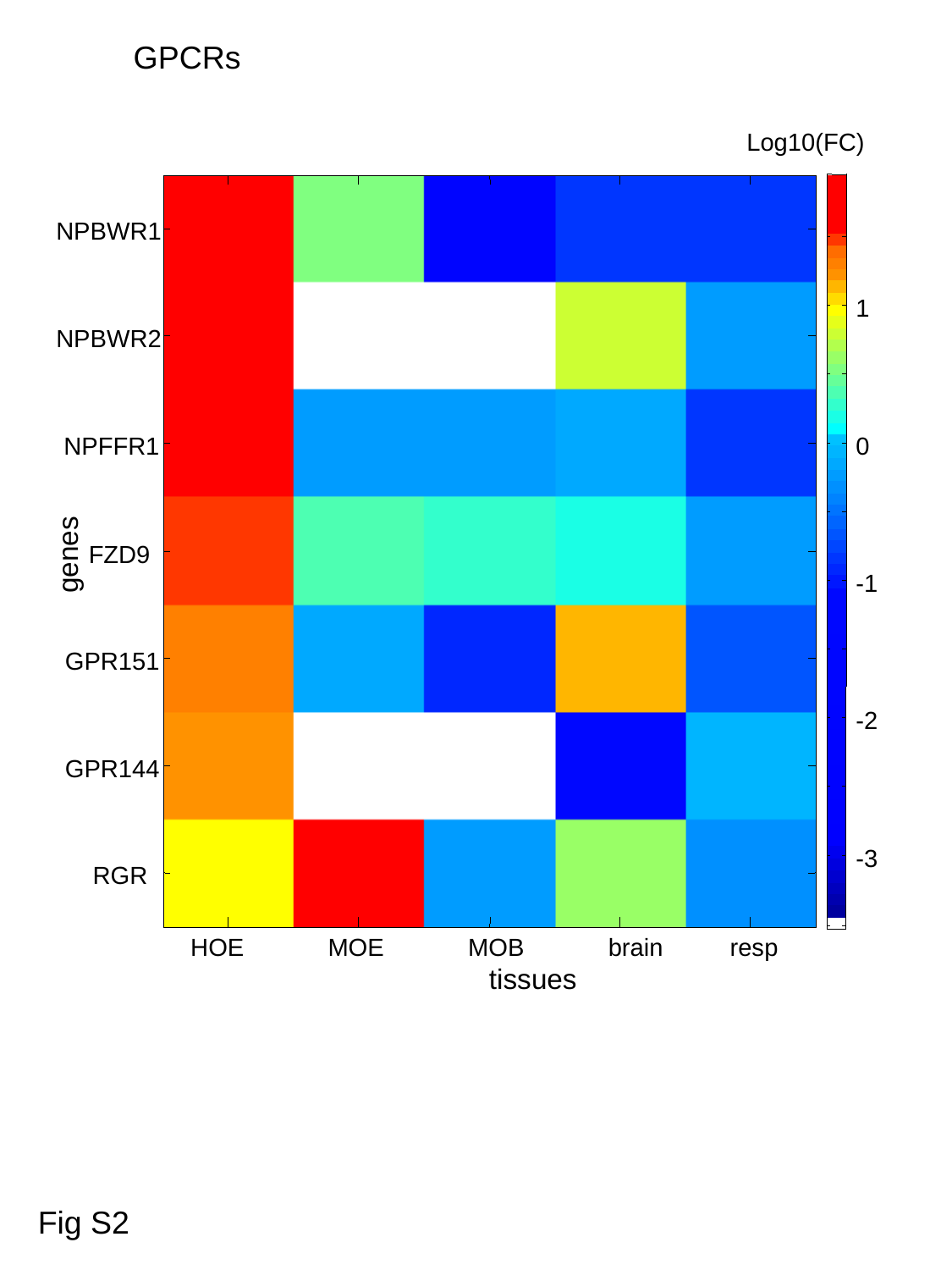

GPCRs
Log10(FC)
NPBWR1
NPBWR2
NPFFR1
FZD9
GPR151
GPR144
RGR
1
0
-1
-2
-3
genes
HOE
MOE
MOB
brain
resp
tissues
Fig S2

## Slide 4
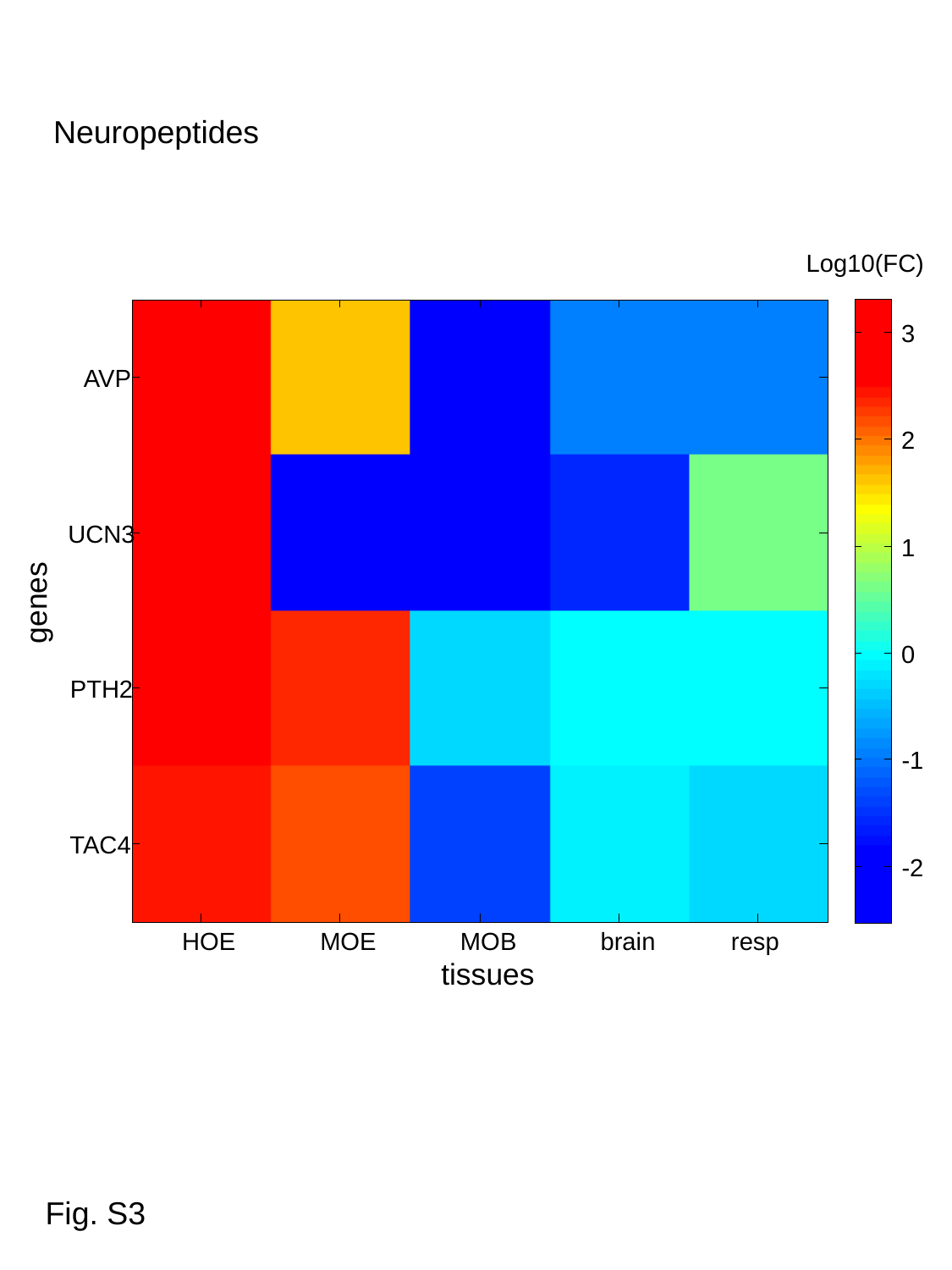

Neuropeptides
Log10(FC)
3
AVP
2
UCN3
1
genes
0
PTH2
-1
TAC4
-2
HOE
MOE
MOB
brain
resp
tissues
Fig. S3

## Slide 5
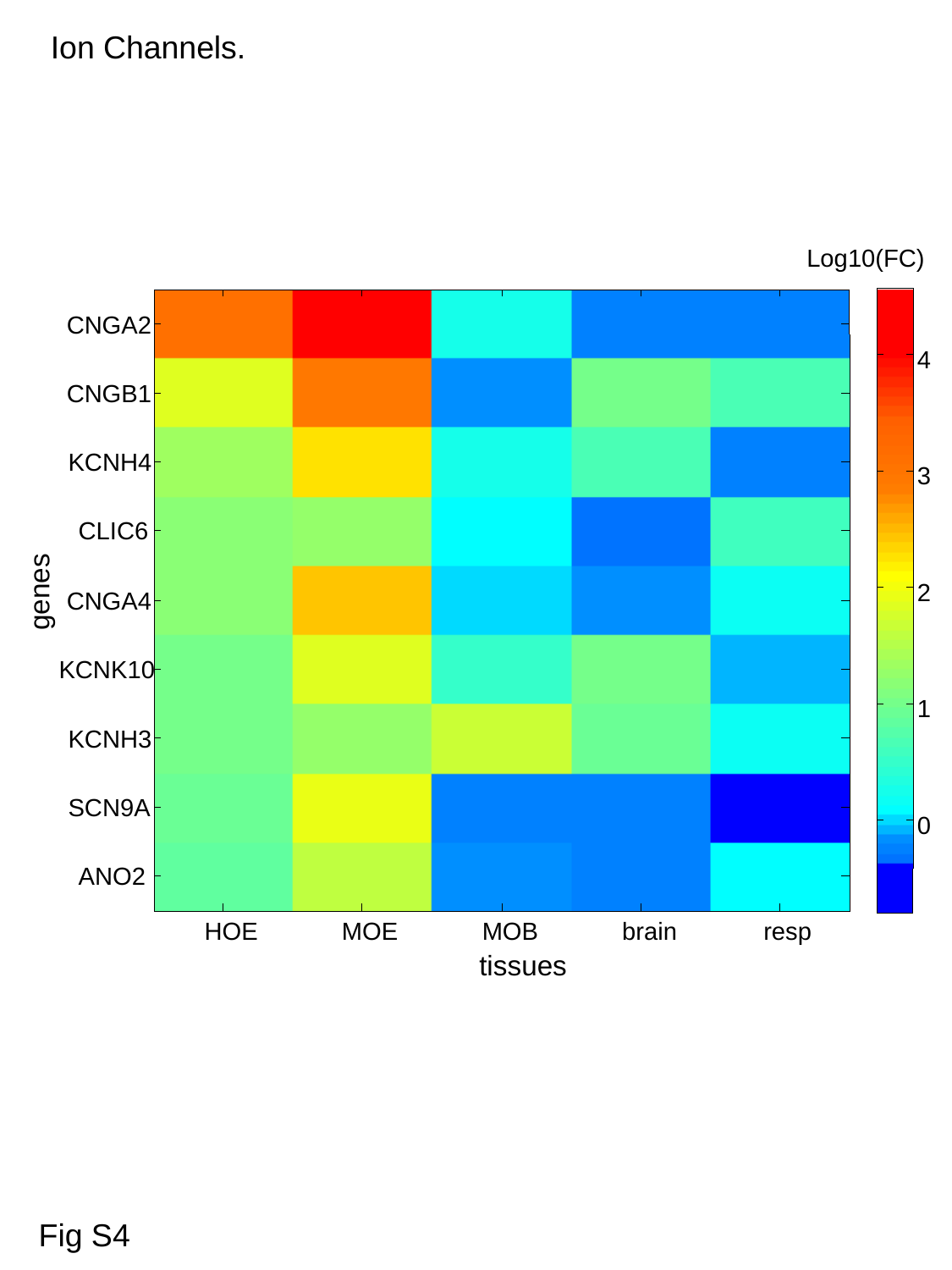

Ion Channels.
Log10(FC)
CNGA2
4
CNGB1
KCNH4
3
CLIC6
genes
2
CNGA4
KCNK10
1
KCNH3
SCN9A
0
ANO2
HOE
MOE
MOB
brain
resp
tissues
Fig S4

## Slide 6
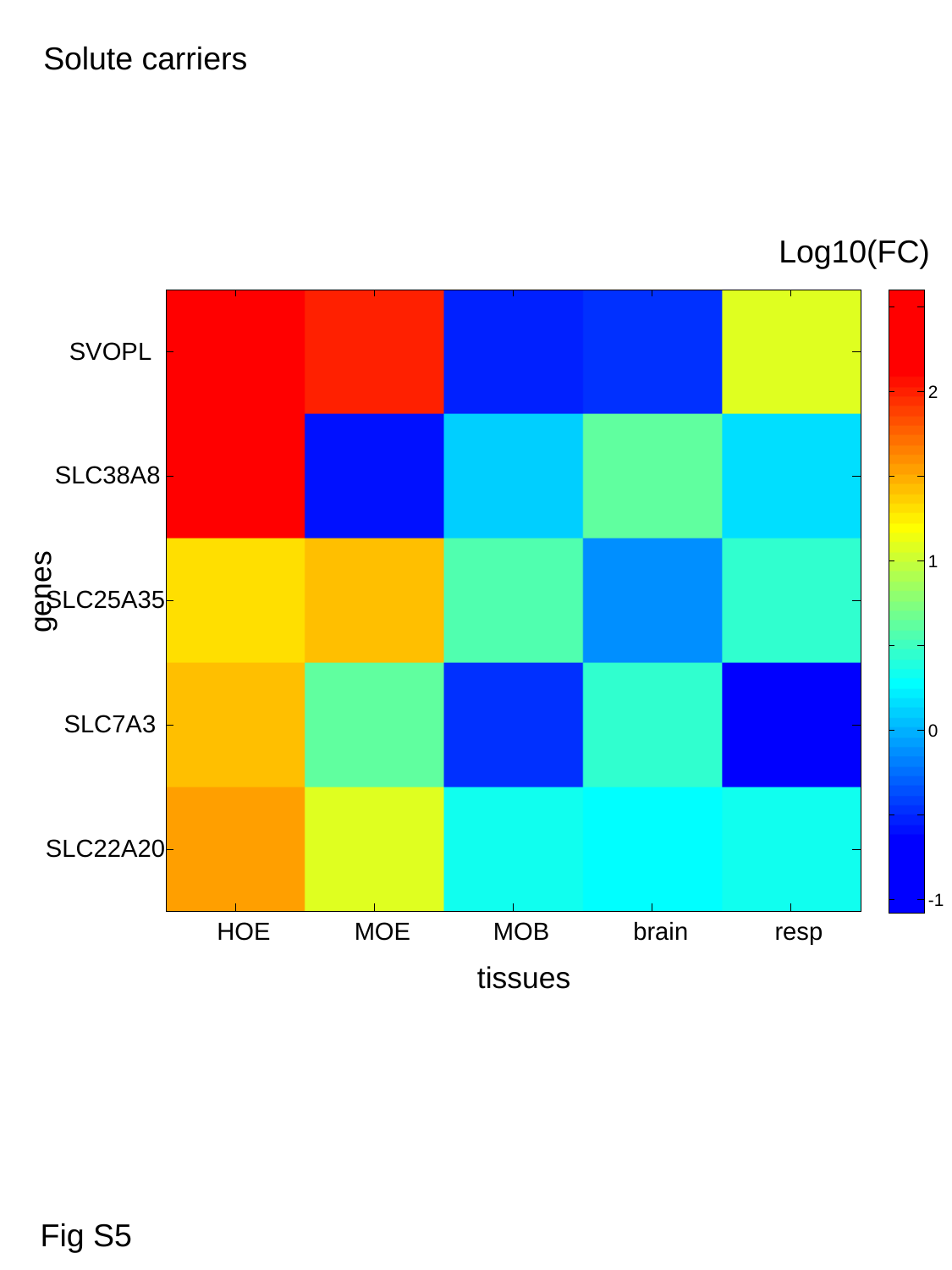

Solute carriers
Log10(FC)
SVOPL
2
SLC38A8
1
genes
SLC25A35
SLC7A3
0
SLC22A20
-1
HOE
MOE
MOB
brain
resp
tissues
Fig S5

## Slide 7
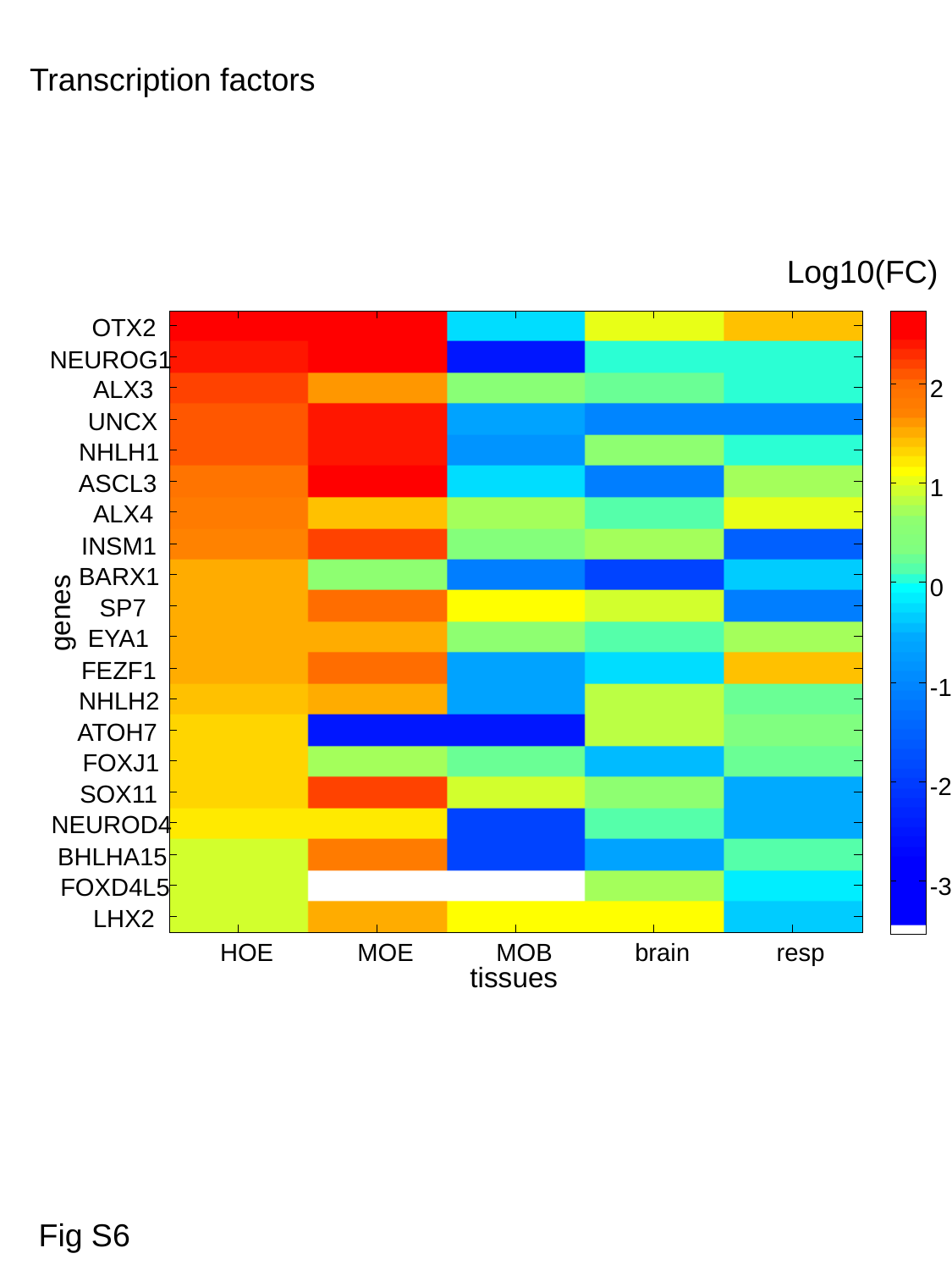

Transcription factors
Log10(FC)
OTX2
NEUROG1
ALX3
UNCX
NHLH1
ASCL3
ALX4
INSM1
BARX1
SP7
EYA1
FEZF1
NHLH2
ATOH7
FOXJ1
SOX11
NEUROD4
BHLHA15
FOXD4L5
LHX2
2
1
0
genes
-1
-2
-3
HOE
MOE
MOB
brain
resp
tissues
Fig S6

## Slide 8
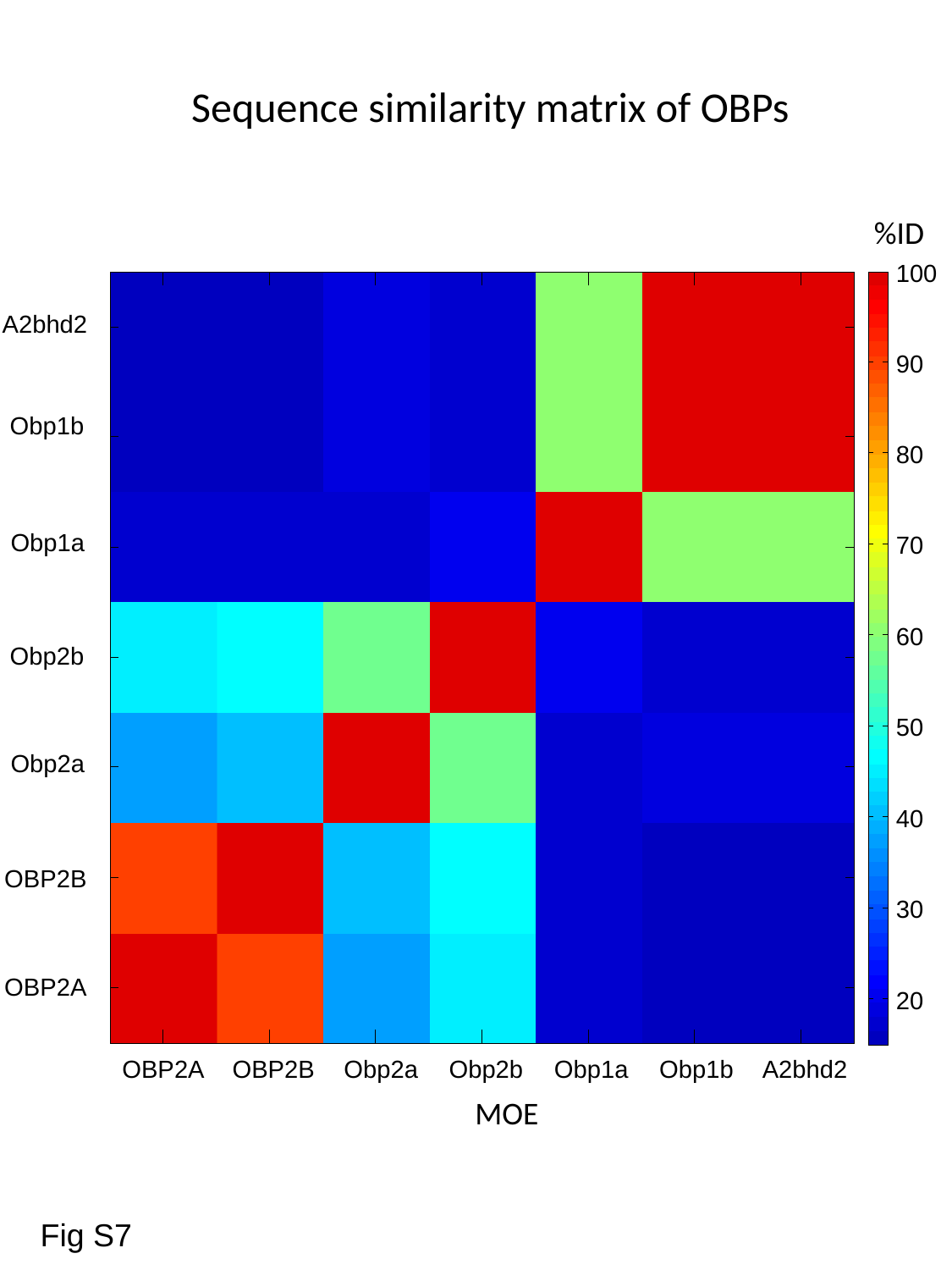

Sequence similarity matrix of OBPs
%ID
100
90
80
70
60
50
40
30
20
A2bhd2
Obp1b
Obp1a
Obp2b
Obp2a
OBP2B
OBP2A
OBP2A
OBP2B
Obp2a
Obp2b
Obp1a
Obp1b
A2bhd2
MOE
Fig S7

## Slide 9
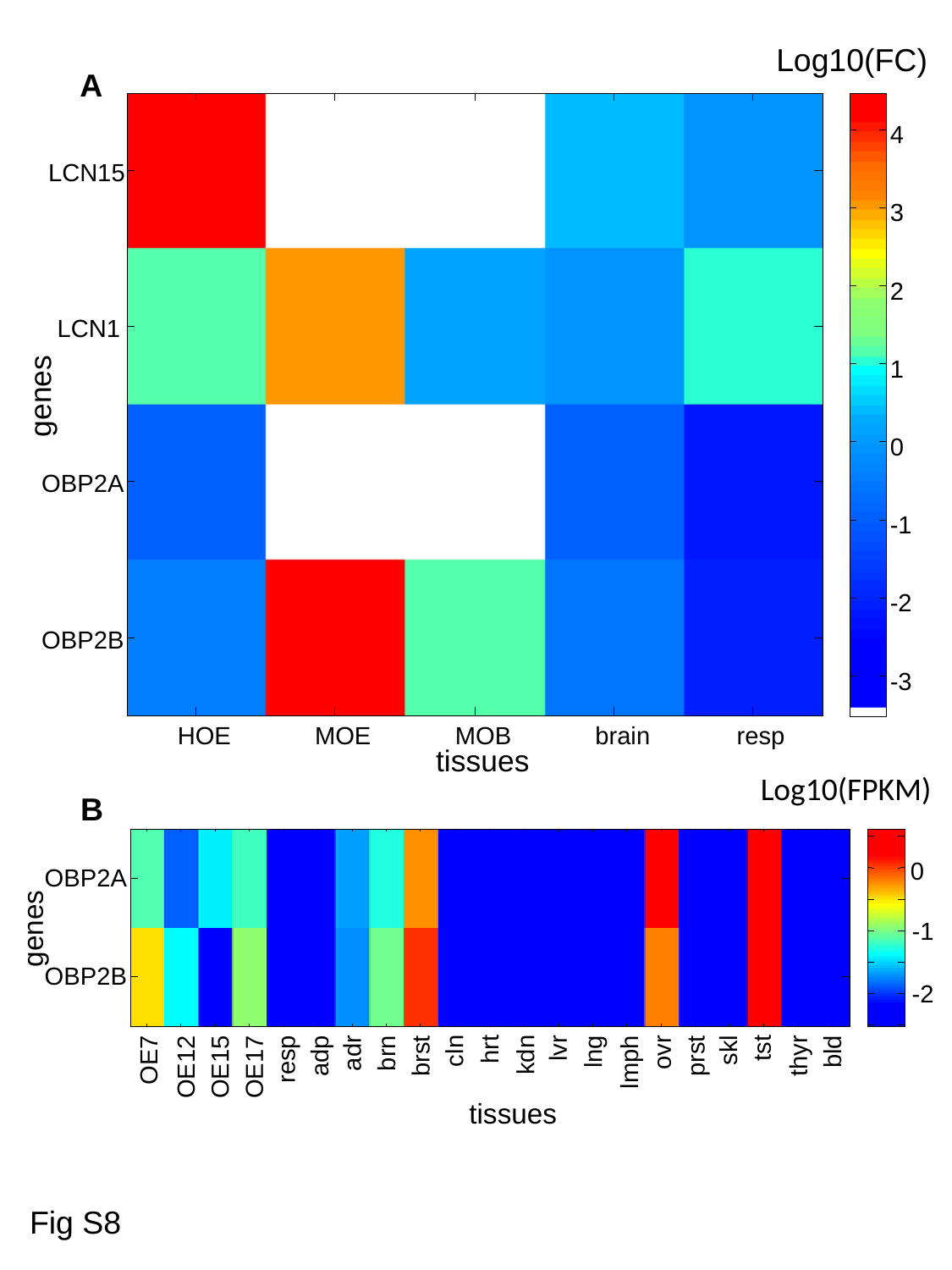

Log10(FC)
A
4
LCN15
LCN1
OBP2A
OBP2B
3
2
1
genes
0
-1
-2
-3
HOE
MOE
MOB
brain
resp
tissues
Log10(FPKM)
B
0
OBP2A
genes
-1
OBP2B
-2
lvr
tst
hrt
skl
cln
lng
bld
ovr
adr
brn
kdn
adp
brst
prst
thyr
resp
OE7
lmph
OE12
OE15
OE17
tissues
Fig S8

## Slide 10
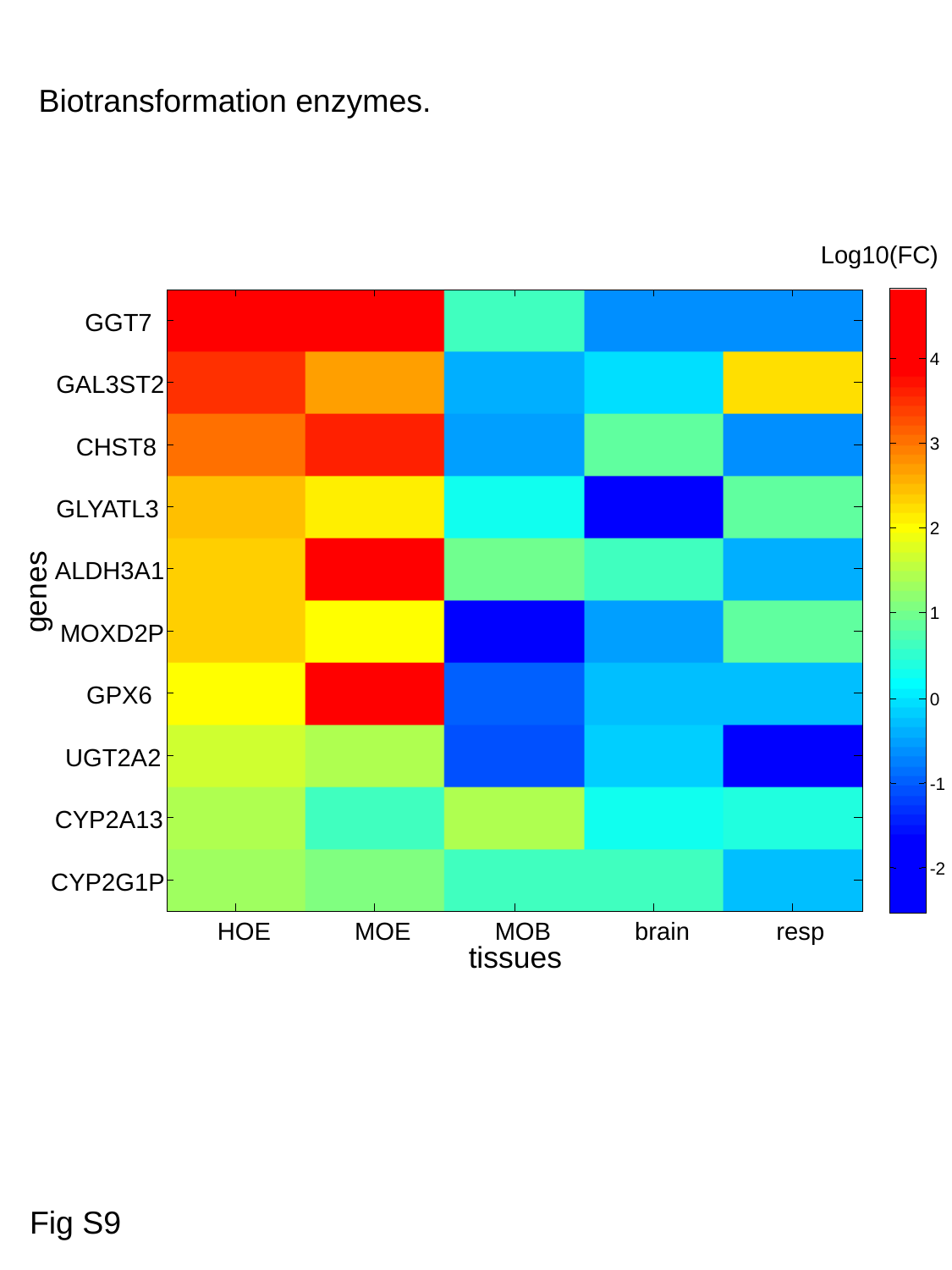

Biotransformation enzymes.
Log10(FC)
GGT7
GAL3ST2
CHST8
GLYATL3
ALDH3A1
MOXD2P
GPX6
UGT2A2
CYP2A13
CYP2G1P
4
3
2
genes
1
0
-1
-2
HOE
MOE
MOB
brain
resp
tissues
Fig S9

## Slide 11
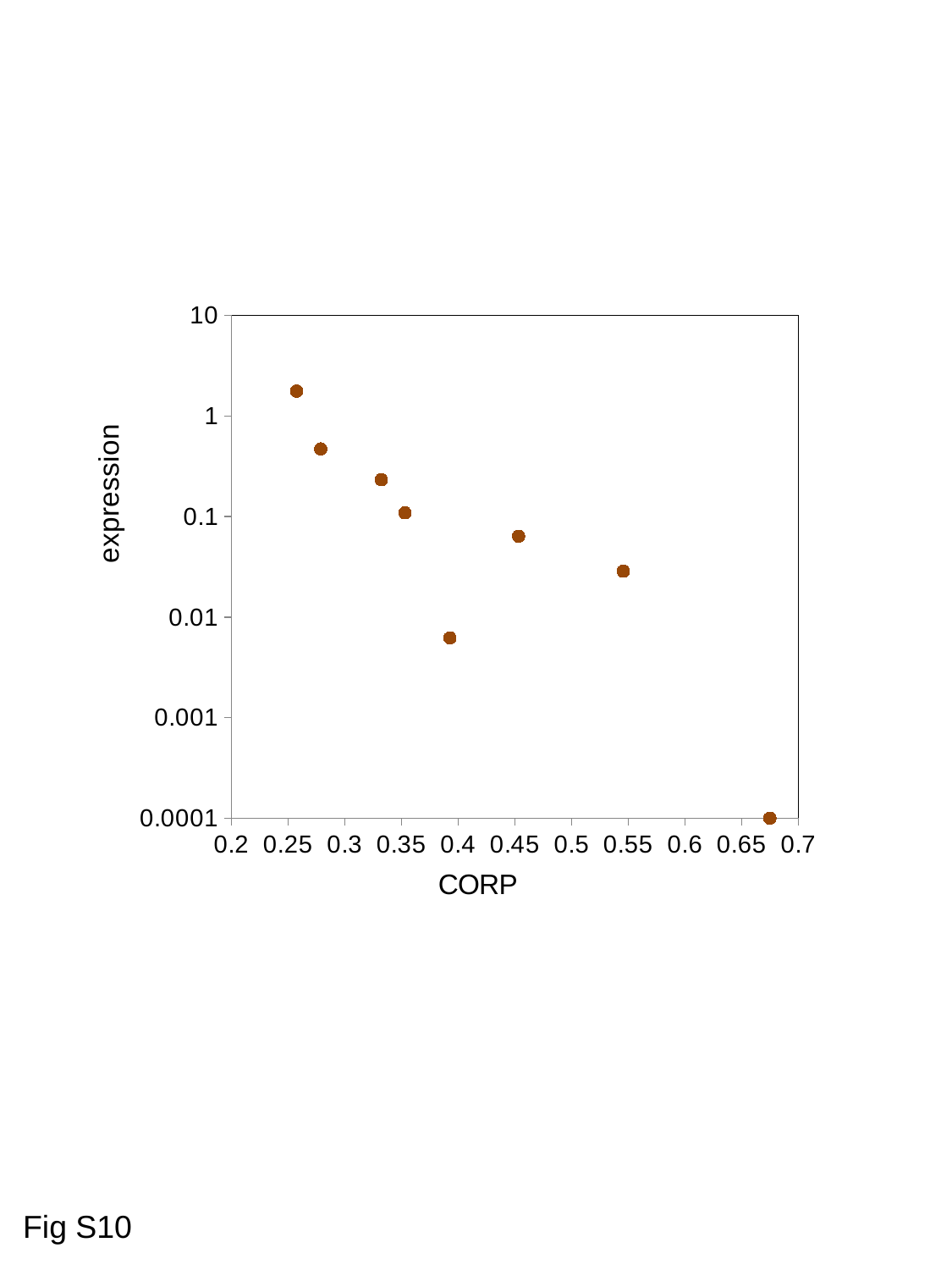

### Chart
| Category | exp |
|---|---|Fig S10

## Slide 12
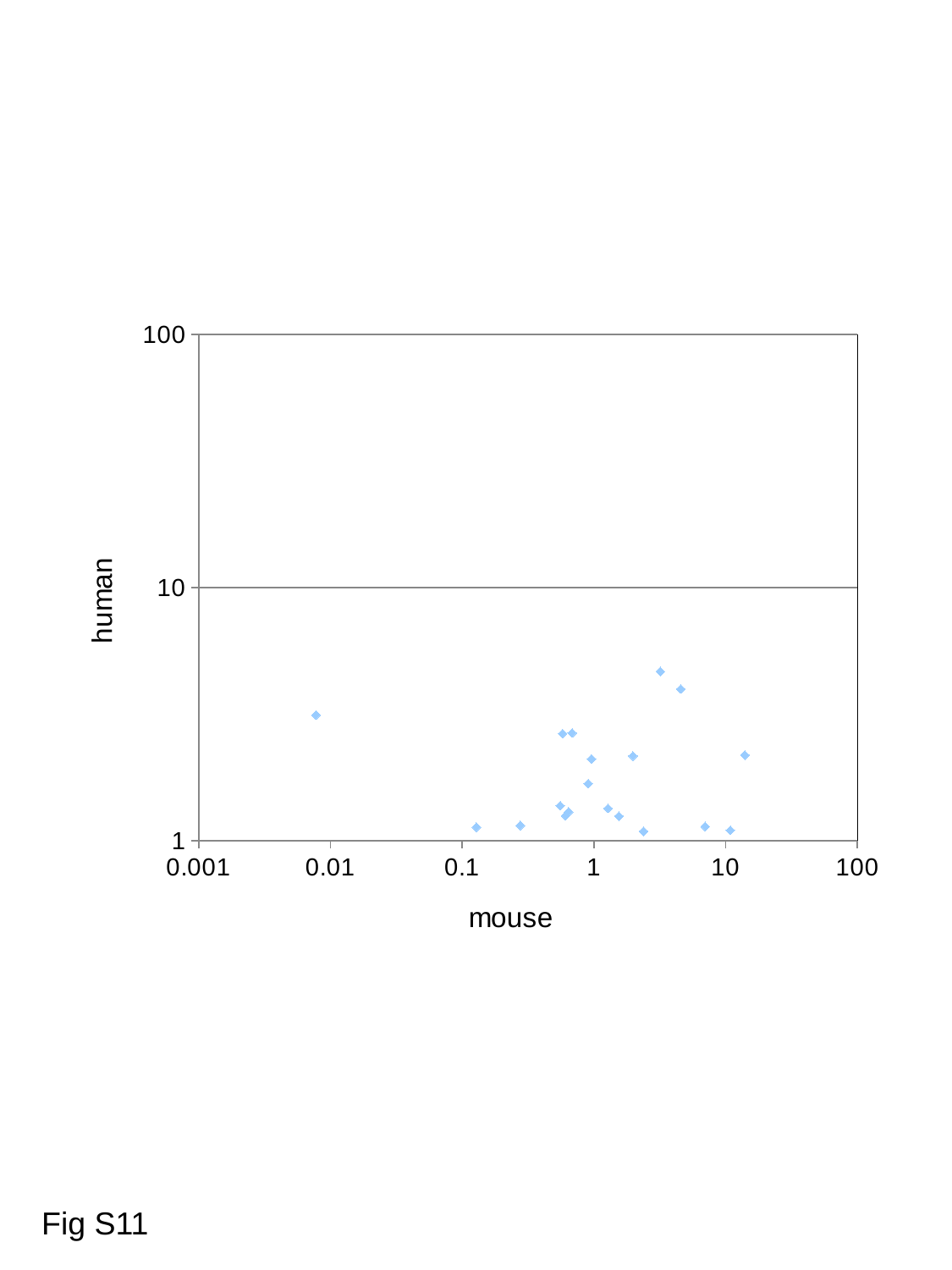

### Chart
| Category | humanFPKM |
|---|---|Fig S11

## Slide 13
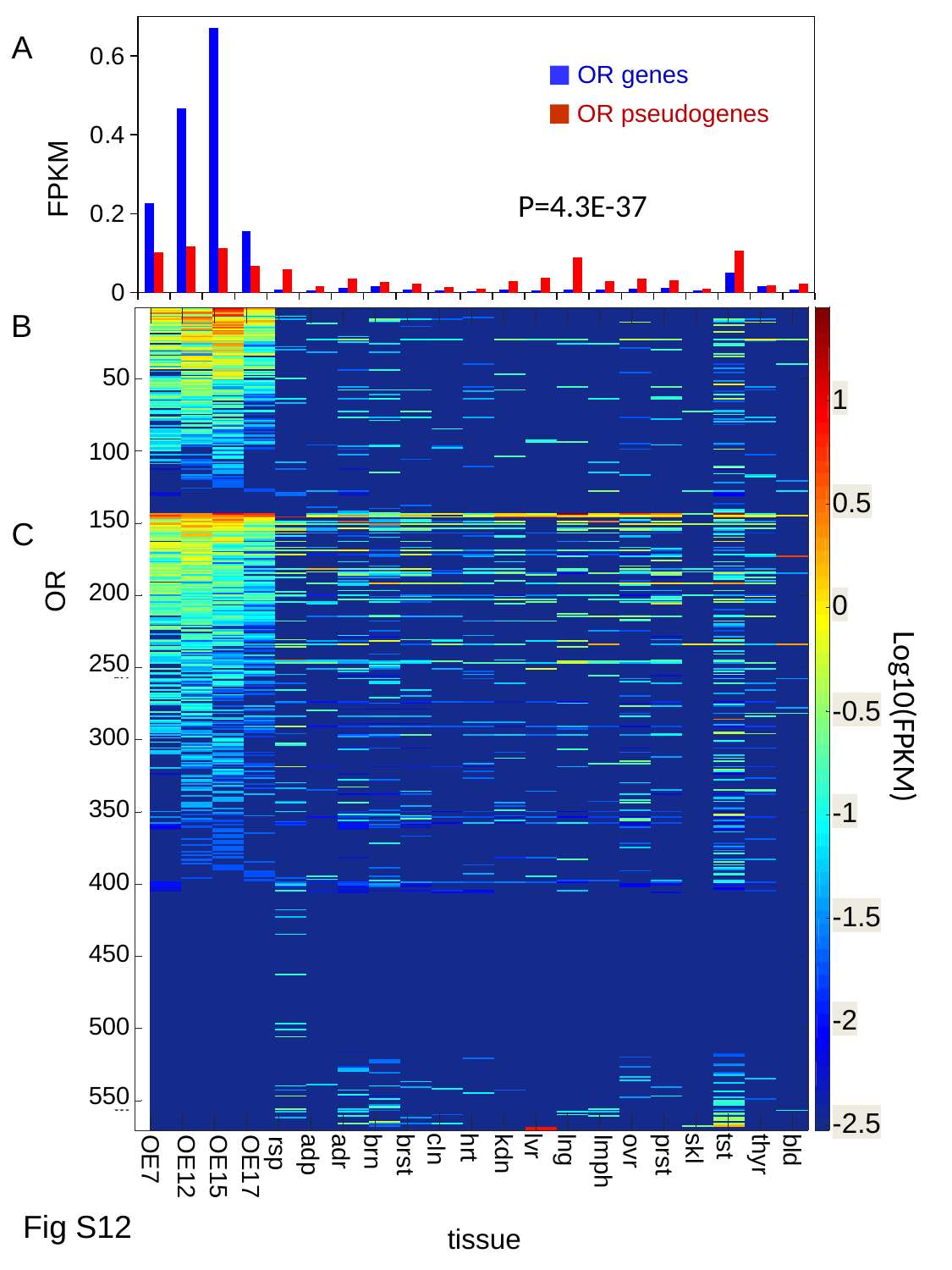

### Chart
| Category | | |
|---|---|---|A
OR genes
OR pseudogenes
FPKM
P=4.3E-37
B
50
100
150
200
250
300
350
400
450
500
550
1
0.5
0
-0.5
-1
-1.5
-2
-2.5
C
OR
Log10(FPKM)
lvr
tst
hrt
skl
cln
lng
bld
ovr
adr
brn
kdn
adp
brst
prst
thyr
rsp
OE7
lmph
OE12
OE15
OE17
Fig S12
tissue

## Slide 14
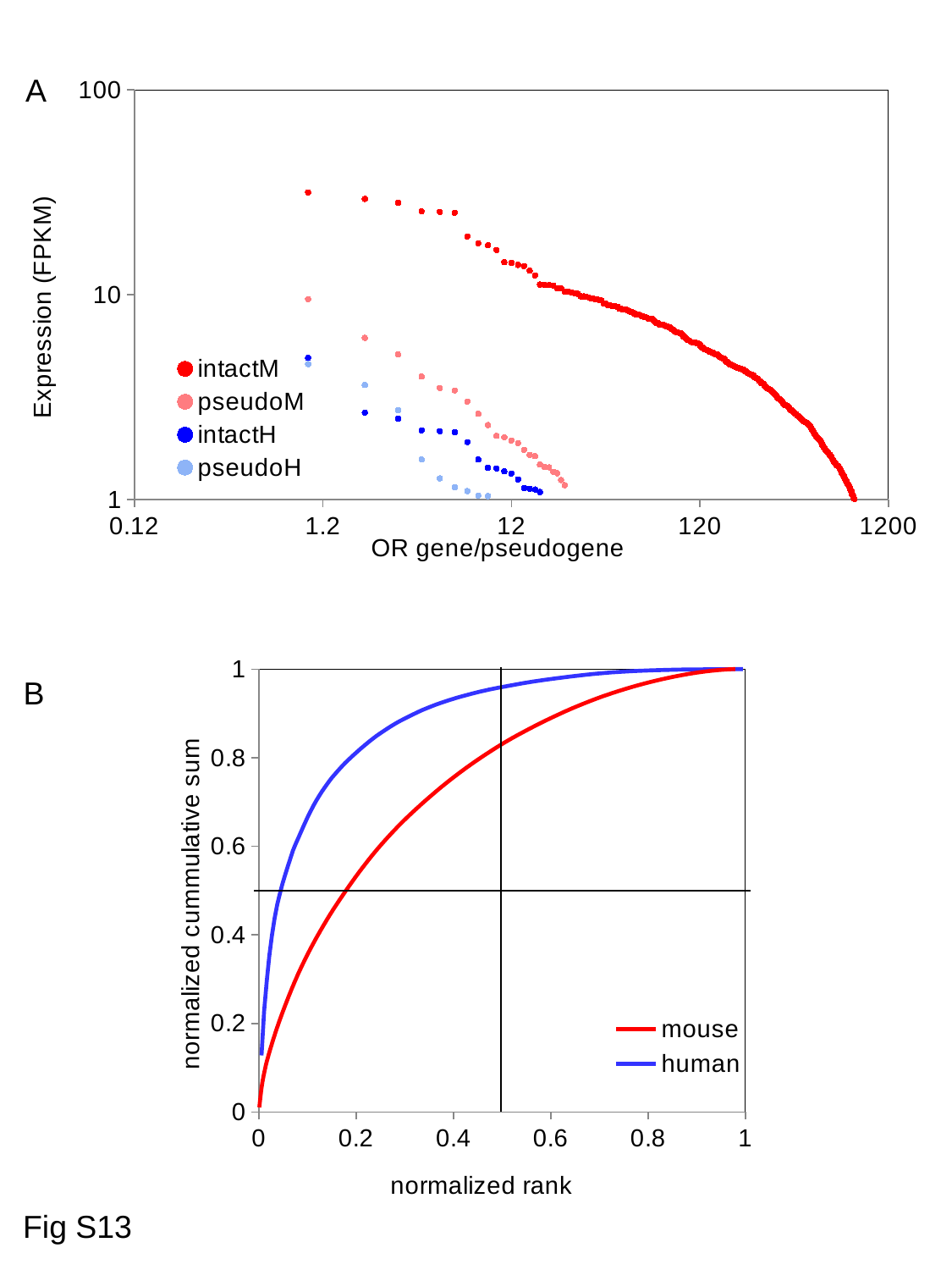

### Chart
| Category | intactM | pseudoM | intactH | pseudoH |
|---|---|---|---|---|A
### Chart
| Category | mouse | |
|---|---|---|B
Fig S13

## Slide 15
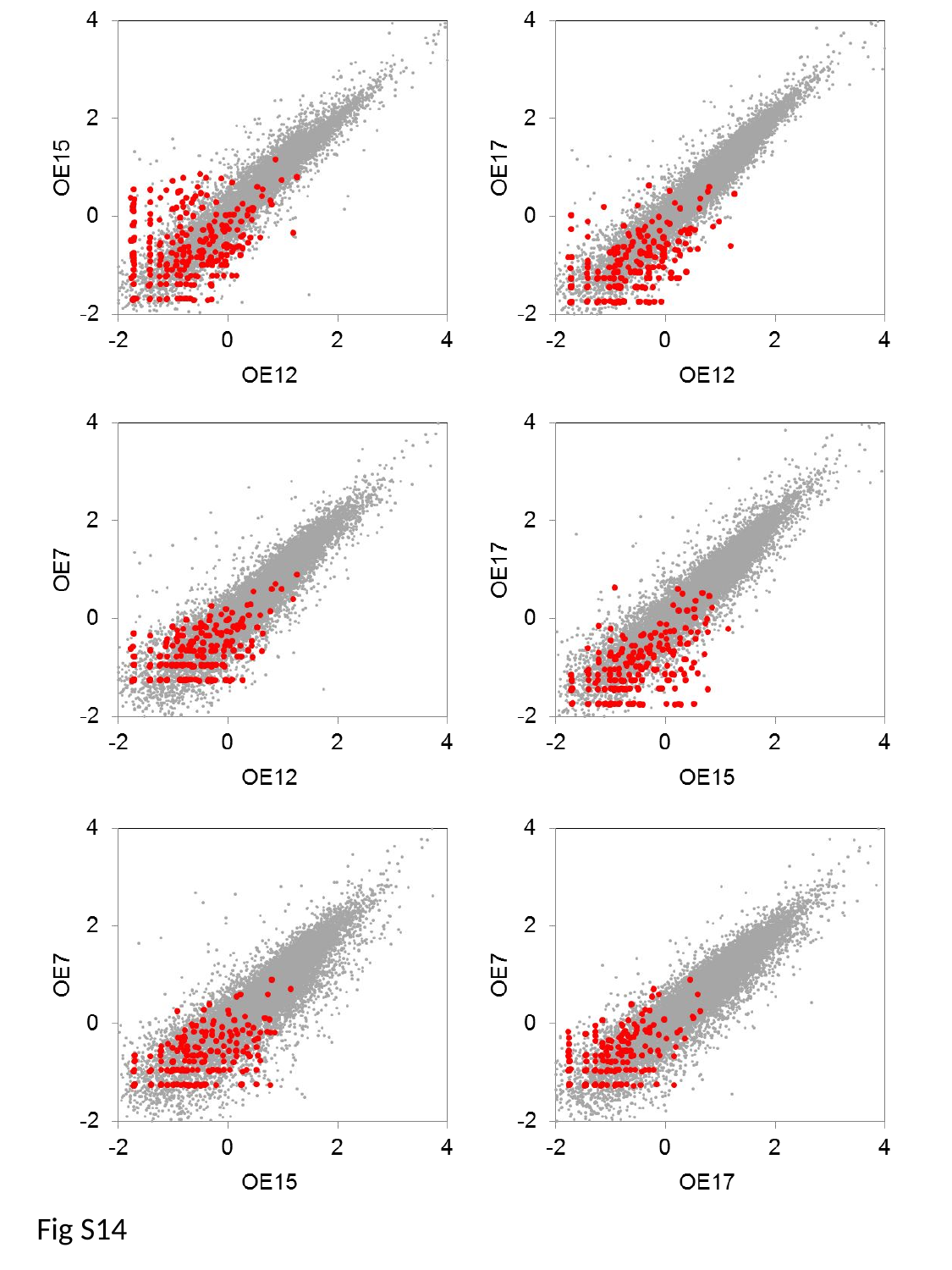

Fig S14

## Slide 16
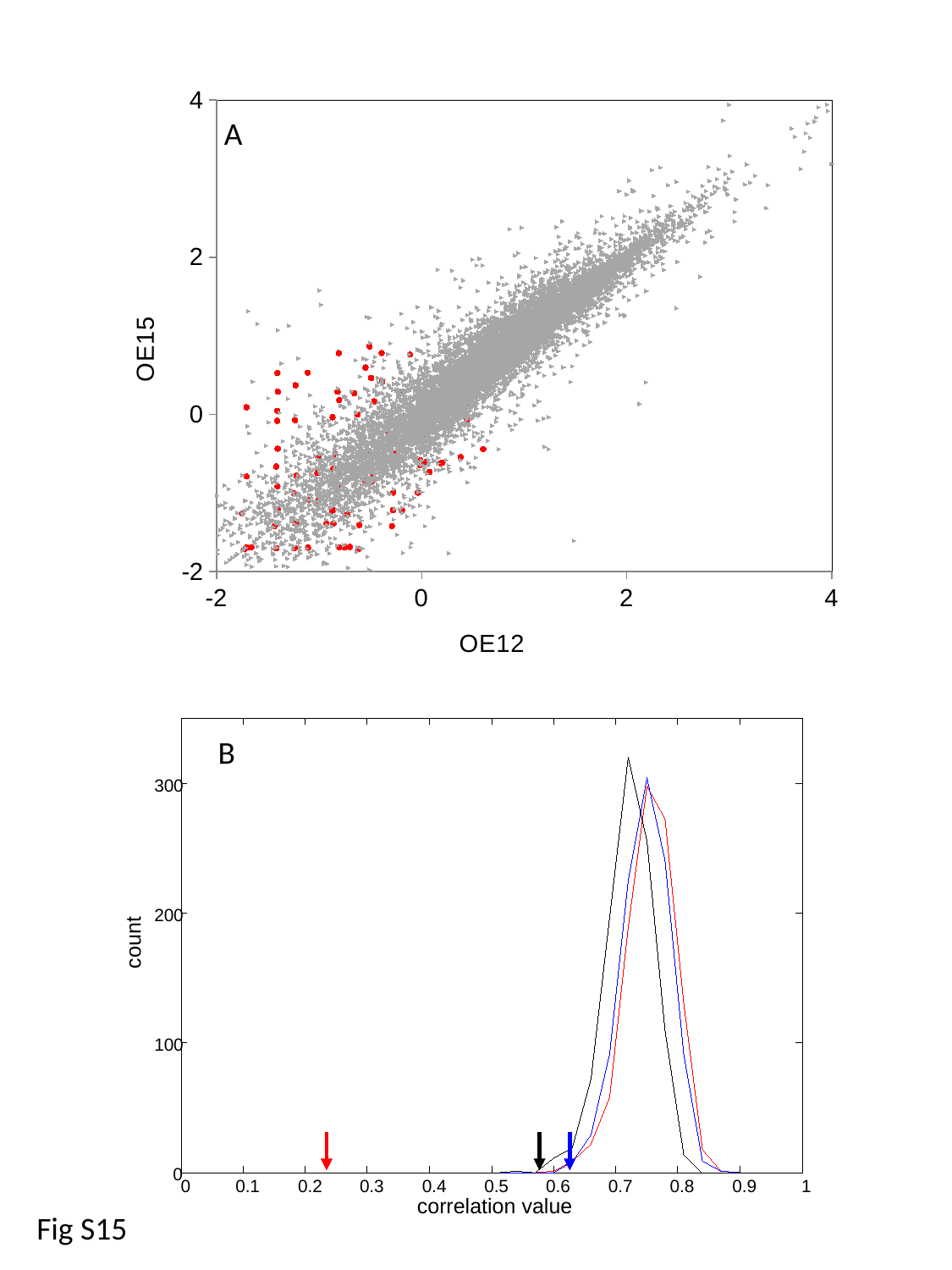

### Chart
| Category | OE7 | |
|---|---|---|A
300
200
count
100
0
0
0.1
0.2
0.3
0.4
0.5
0.6
0.7
0.8
0.9
1
correlation value
B
Fig S15

## Slide 17
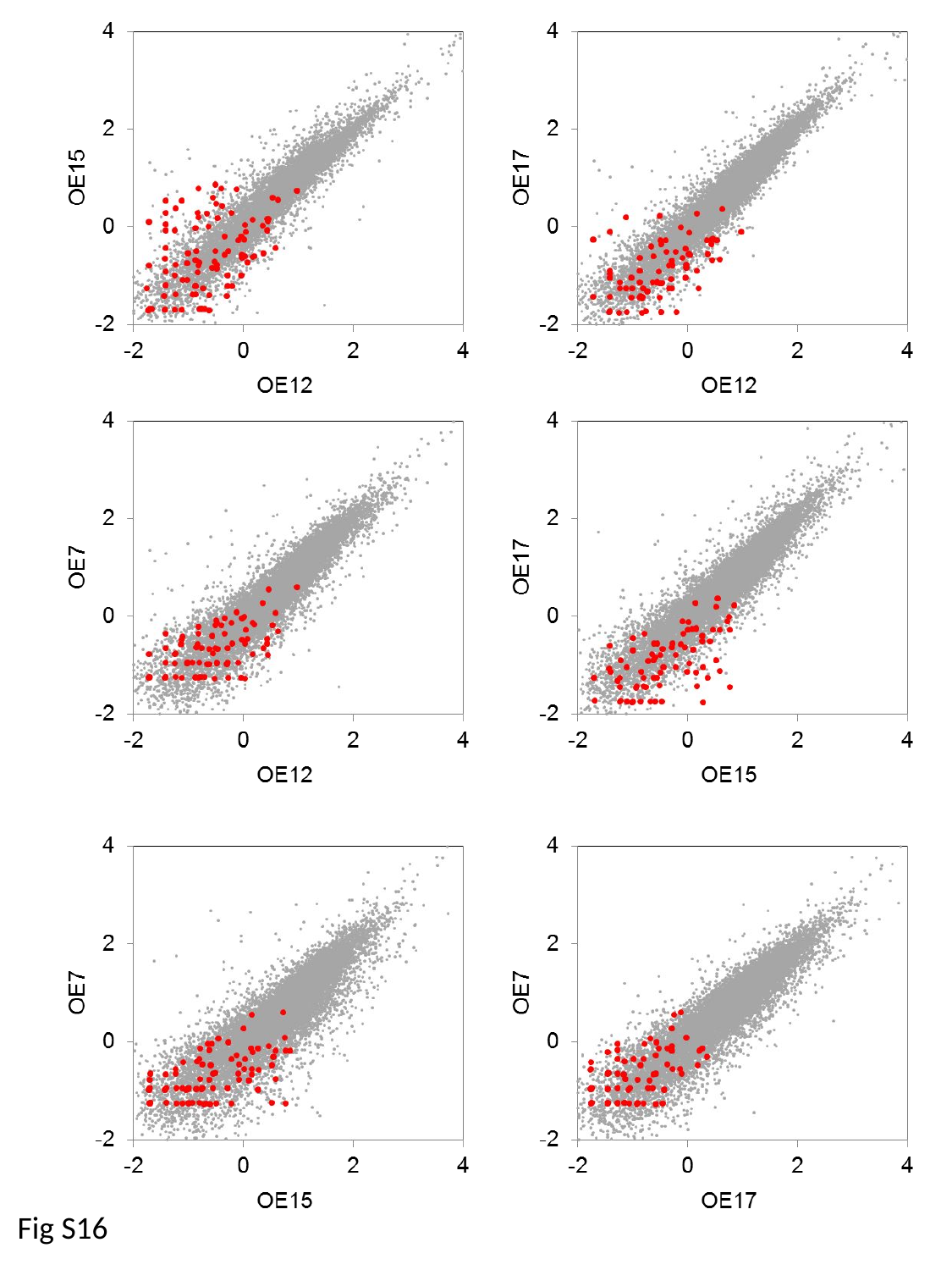

Fig S16

## Slide 18
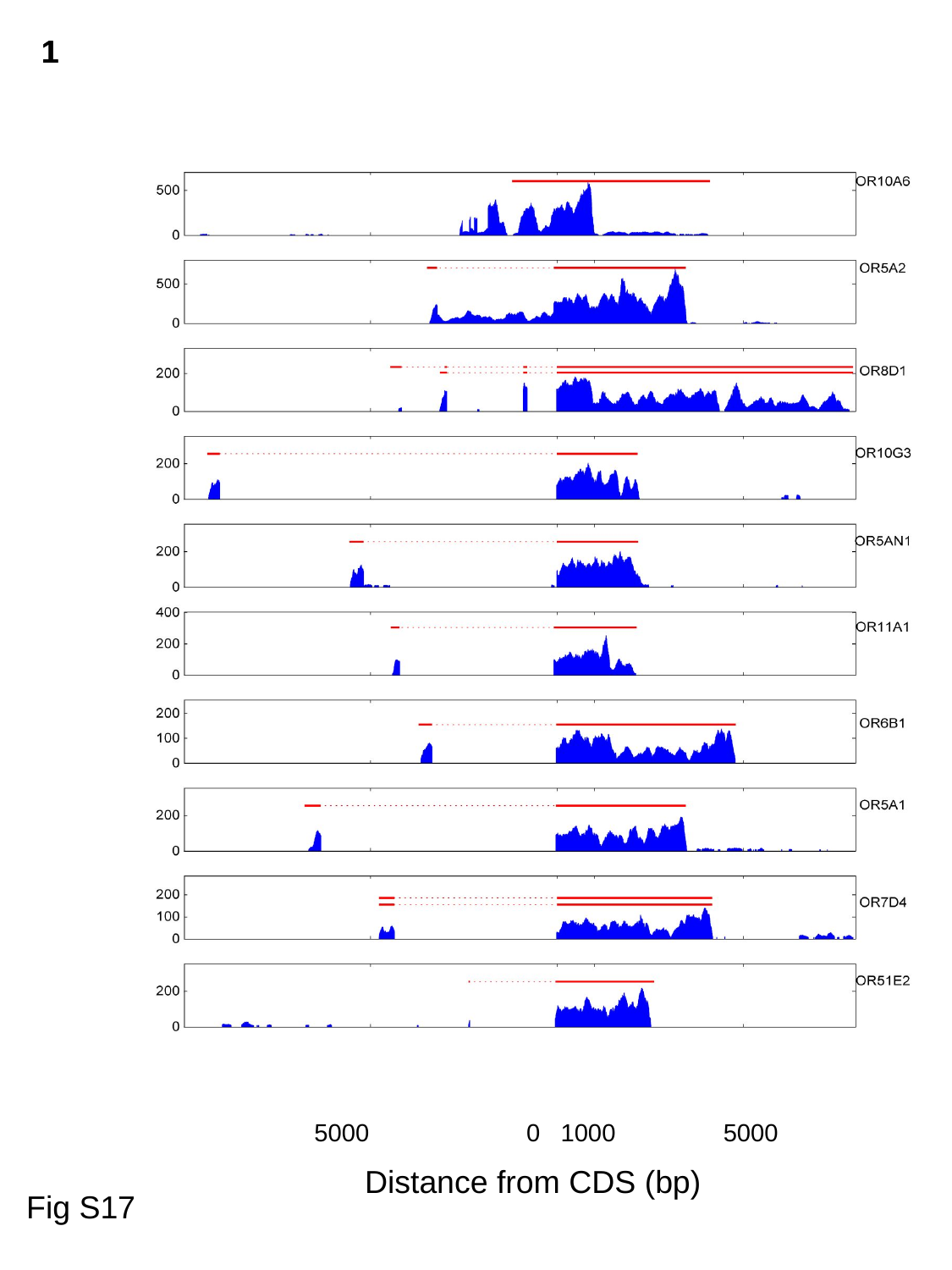

1
1
5000
0
1000
5000
Distance from CDS (bp)
Fig S17

## Slide 19
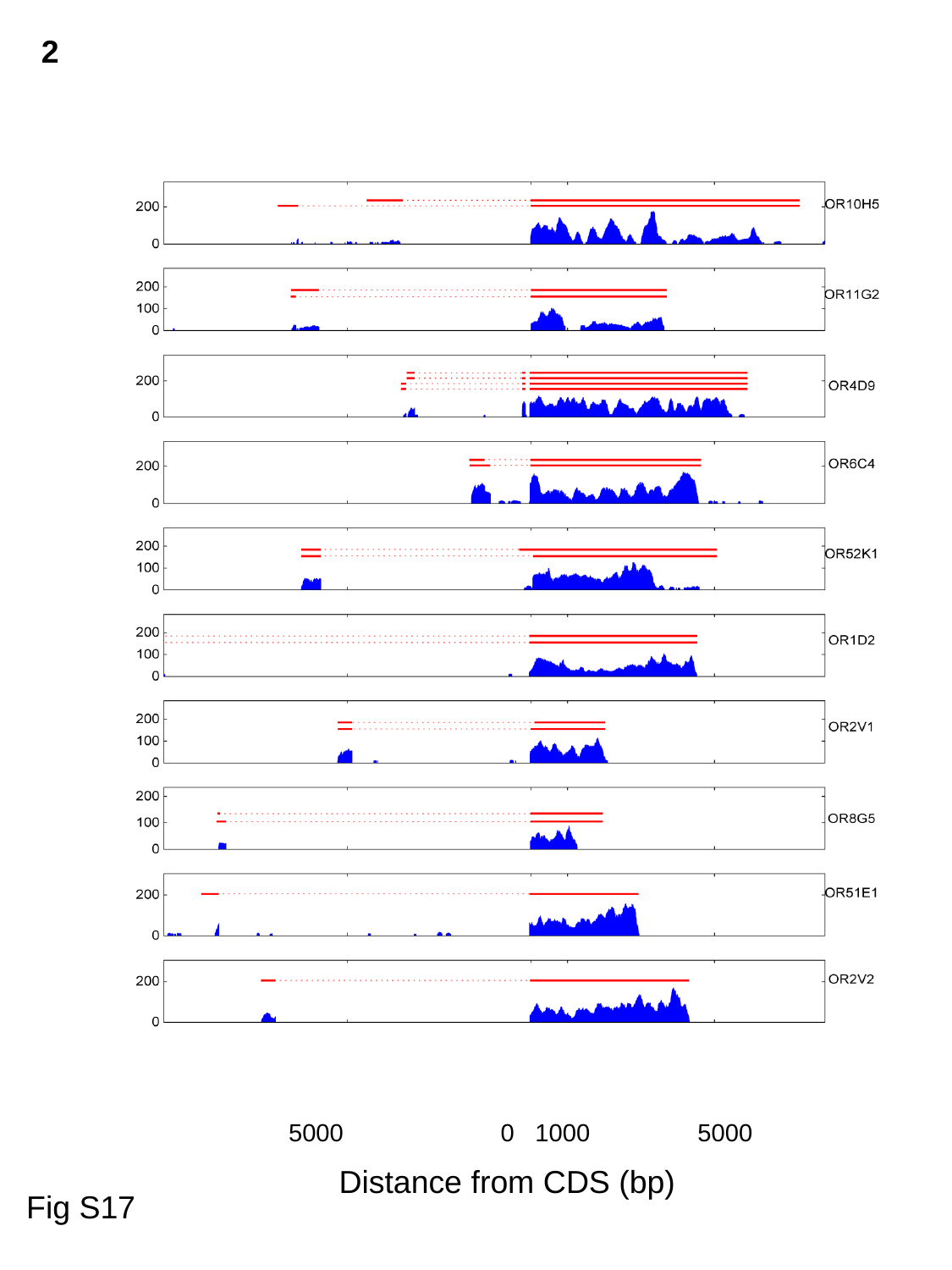

2
5000
0
1000
5000
Distance from CDS (bp)
Fig S17

## Slide 20
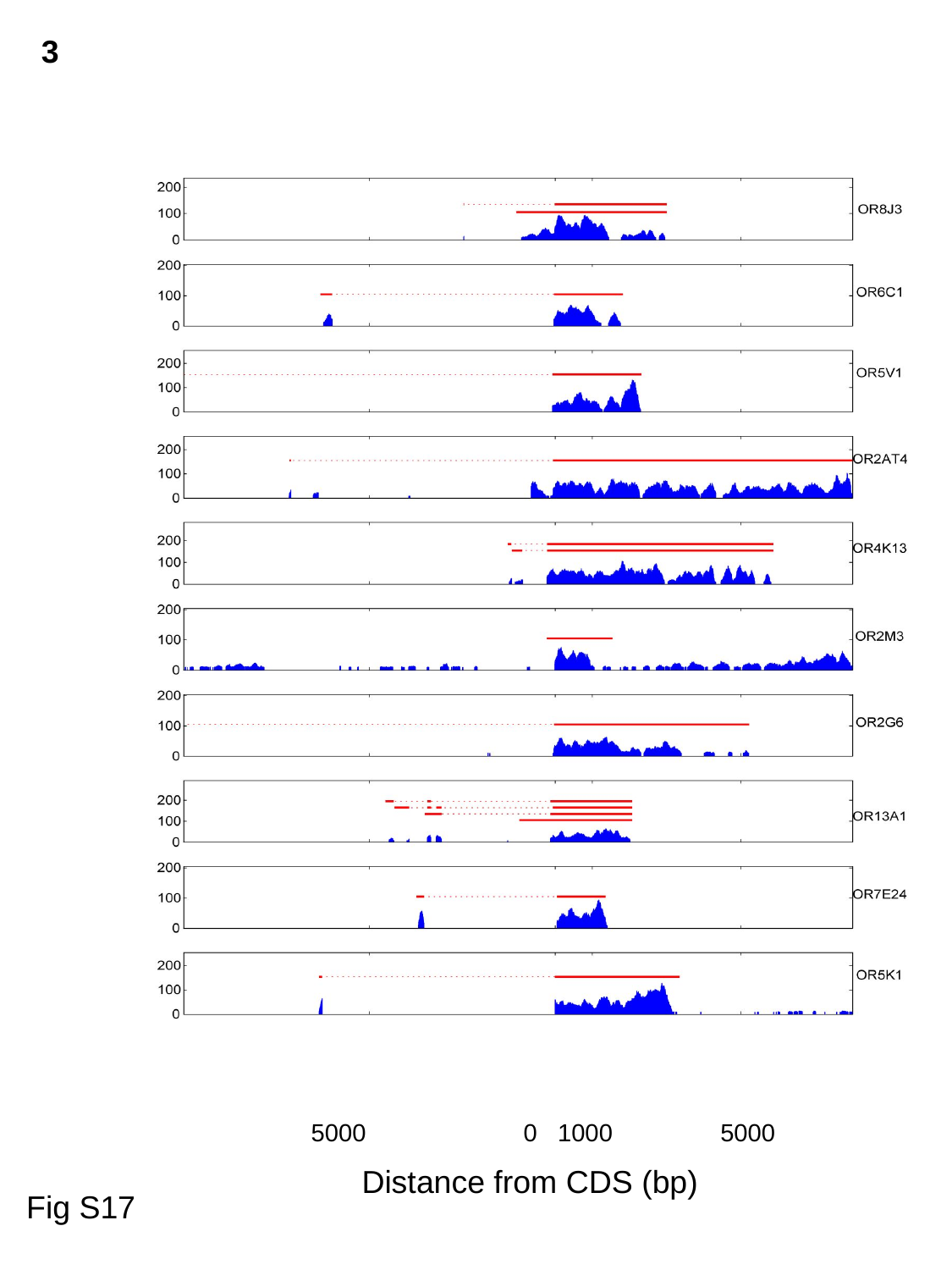

3
5000
0
1000
5000
Distance from CDS (bp)
Fig S17

## Slide 21
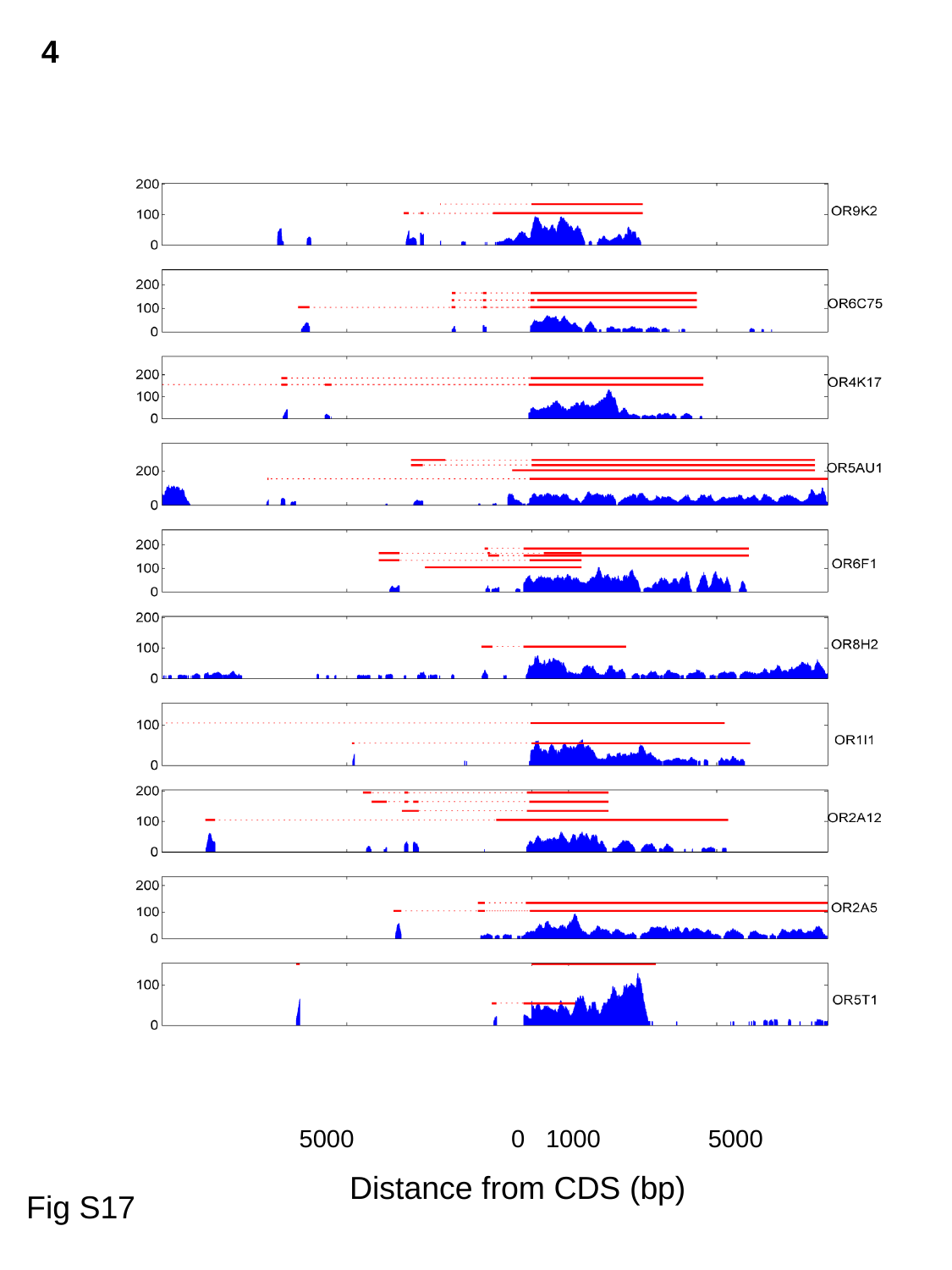

4
5000
0
1000
5000
Distance from CDS (bp)
Fig S17

## Slide 22
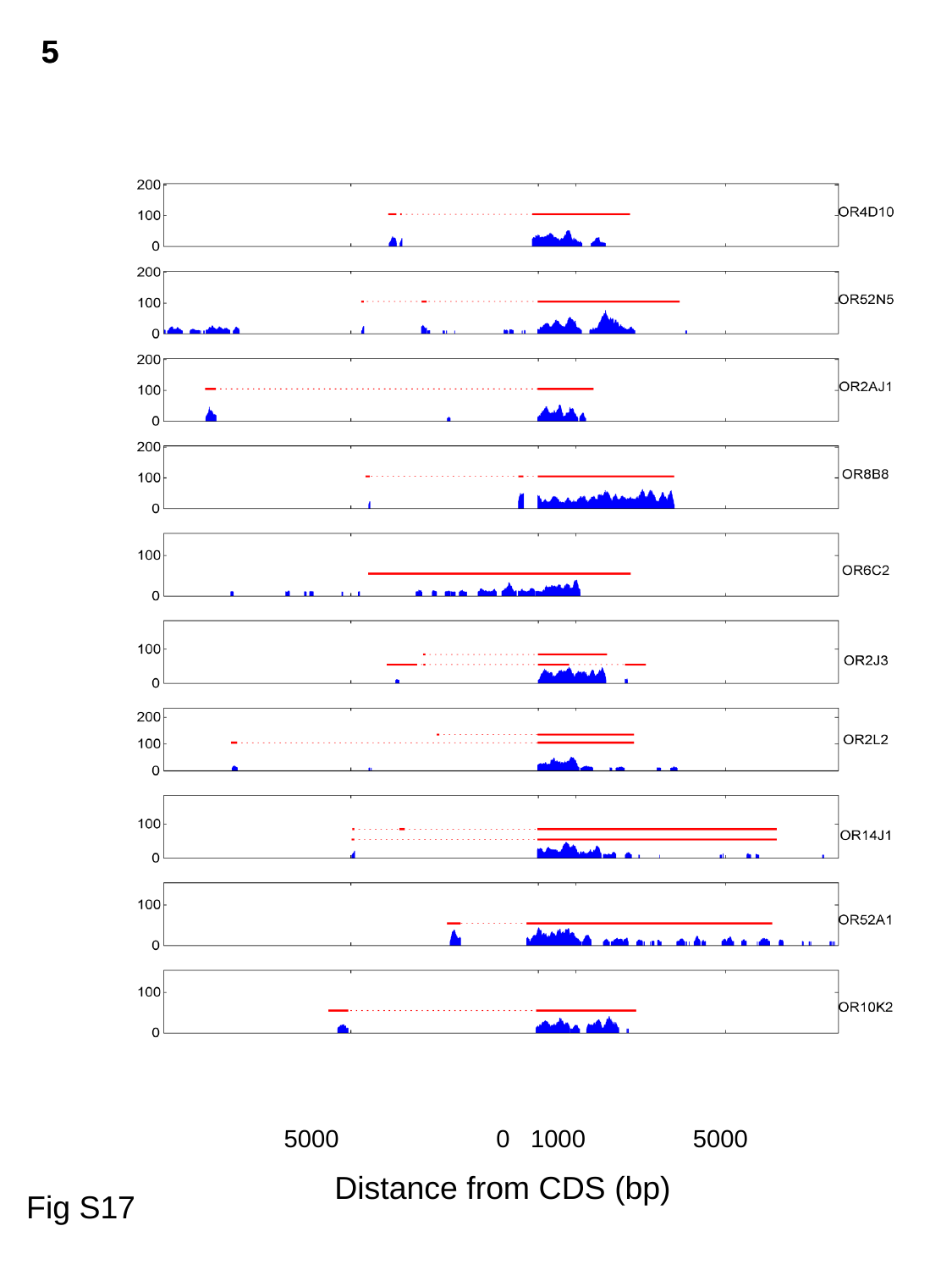

5
5
5000
0
1000
5000
Distance from CDS (bp)
Fig S17

## Slide 23
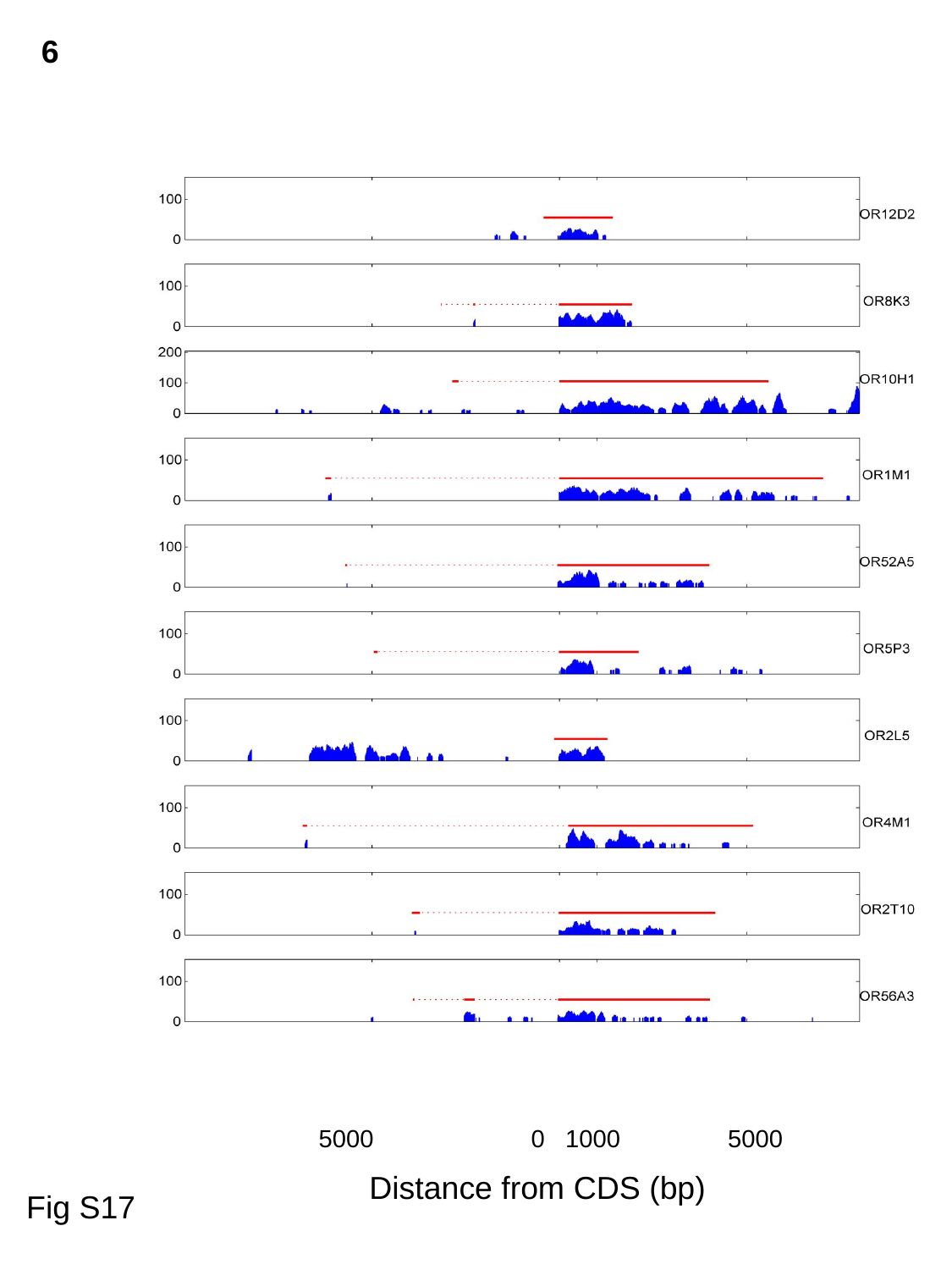

6
5000
0
1000
5000
Distance from CDS (bp)
Fig S17

## Slide 24
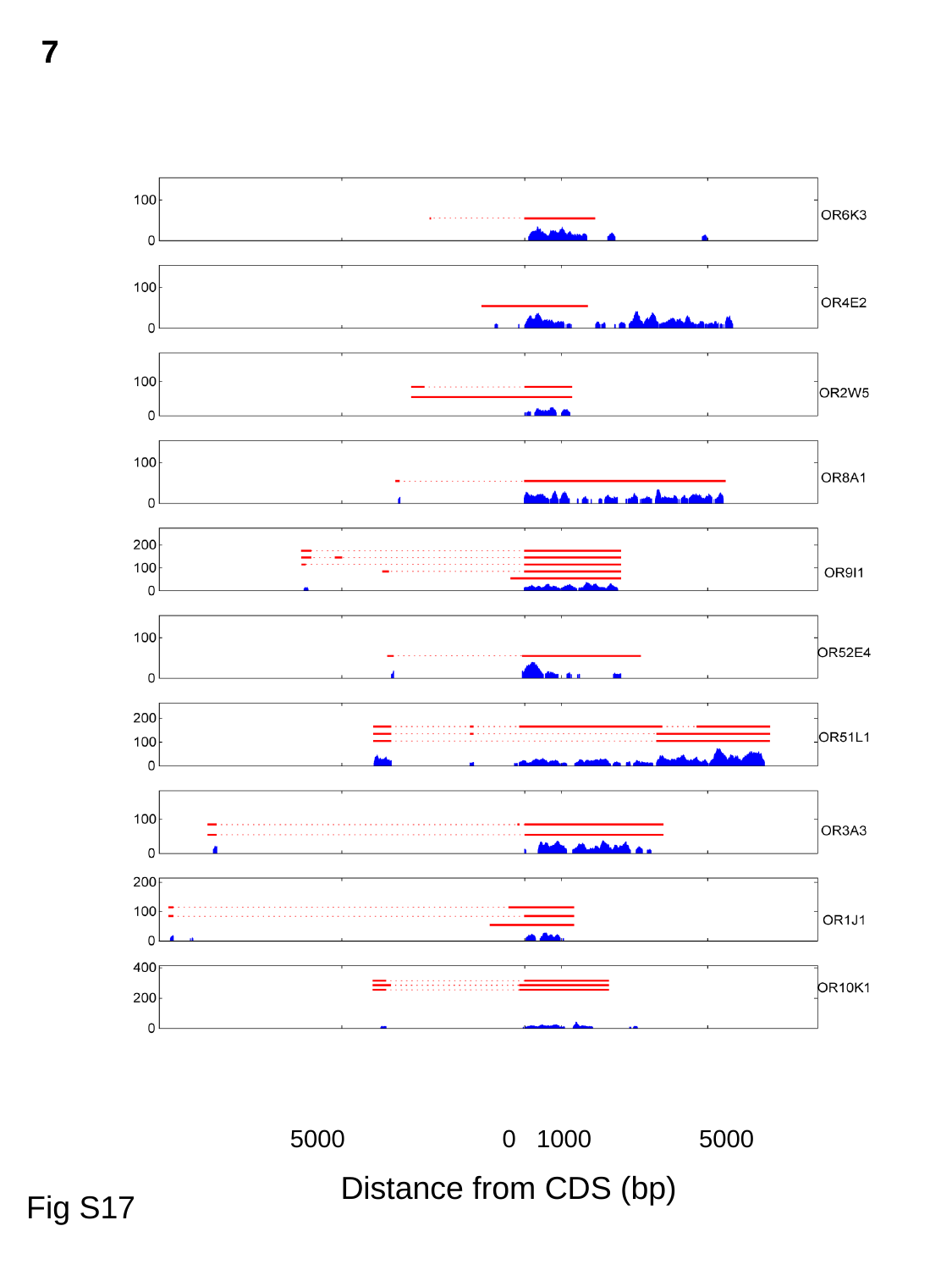

7
7
5000
0
1000
5000
Distance from CDS (bp)
Fig S17

## Slide 25
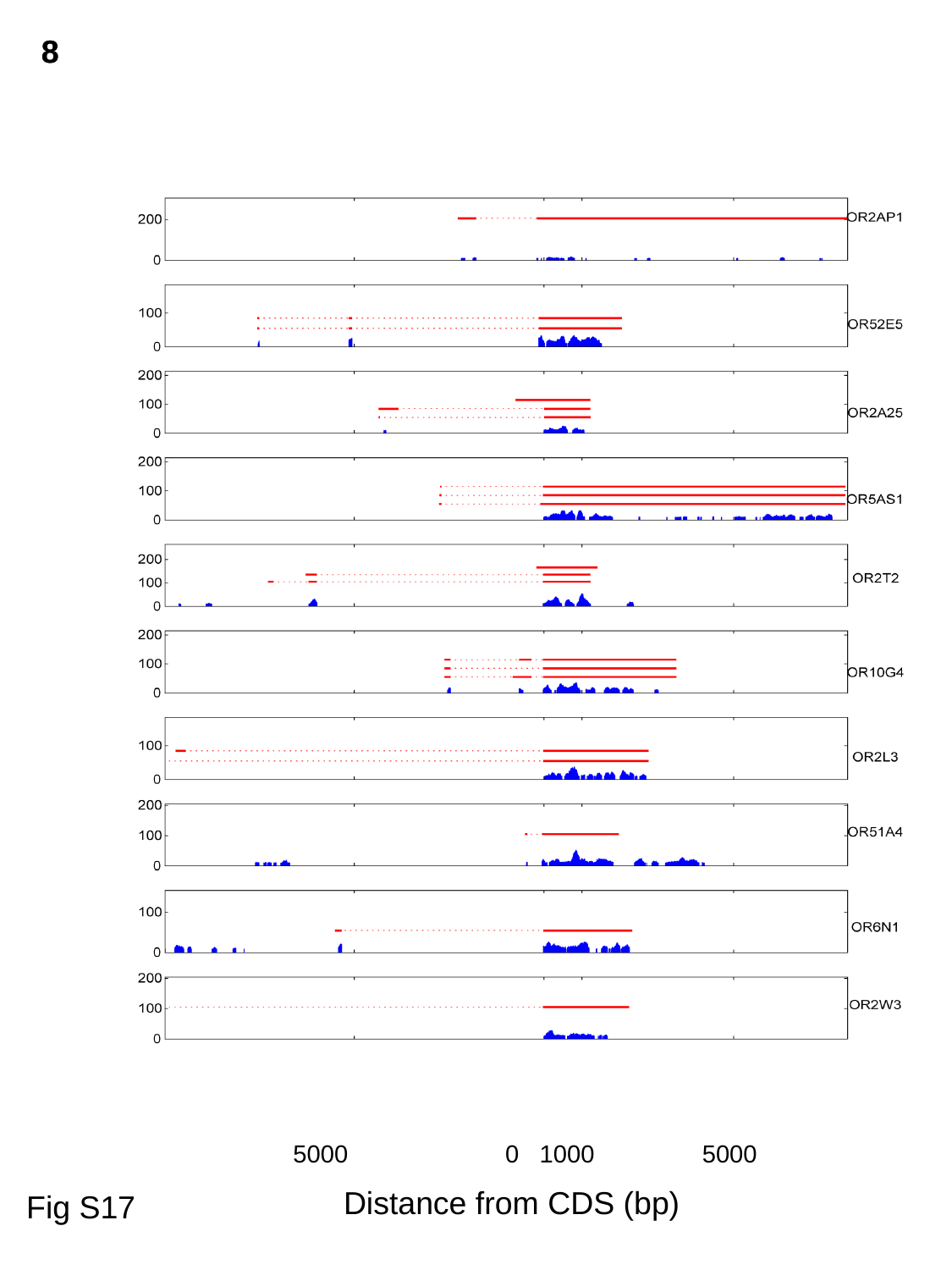

8
8
5000
0
1000
5000
Distance from CDS (bp)
Fig S17

## Slide 26
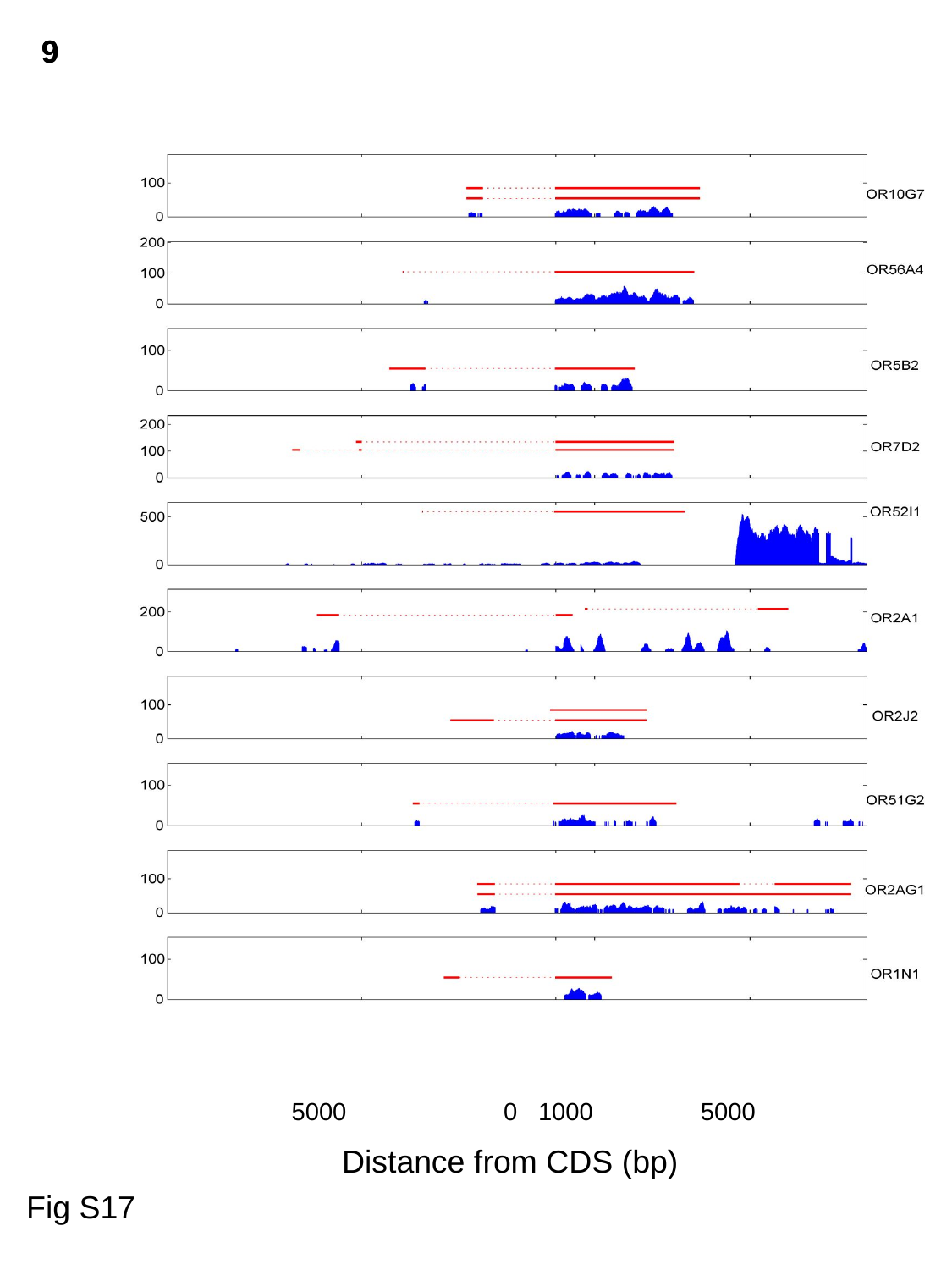

9
9
5000
0
1000
5000
Distance from CDS (bp)
Fig S17

## Slide 27
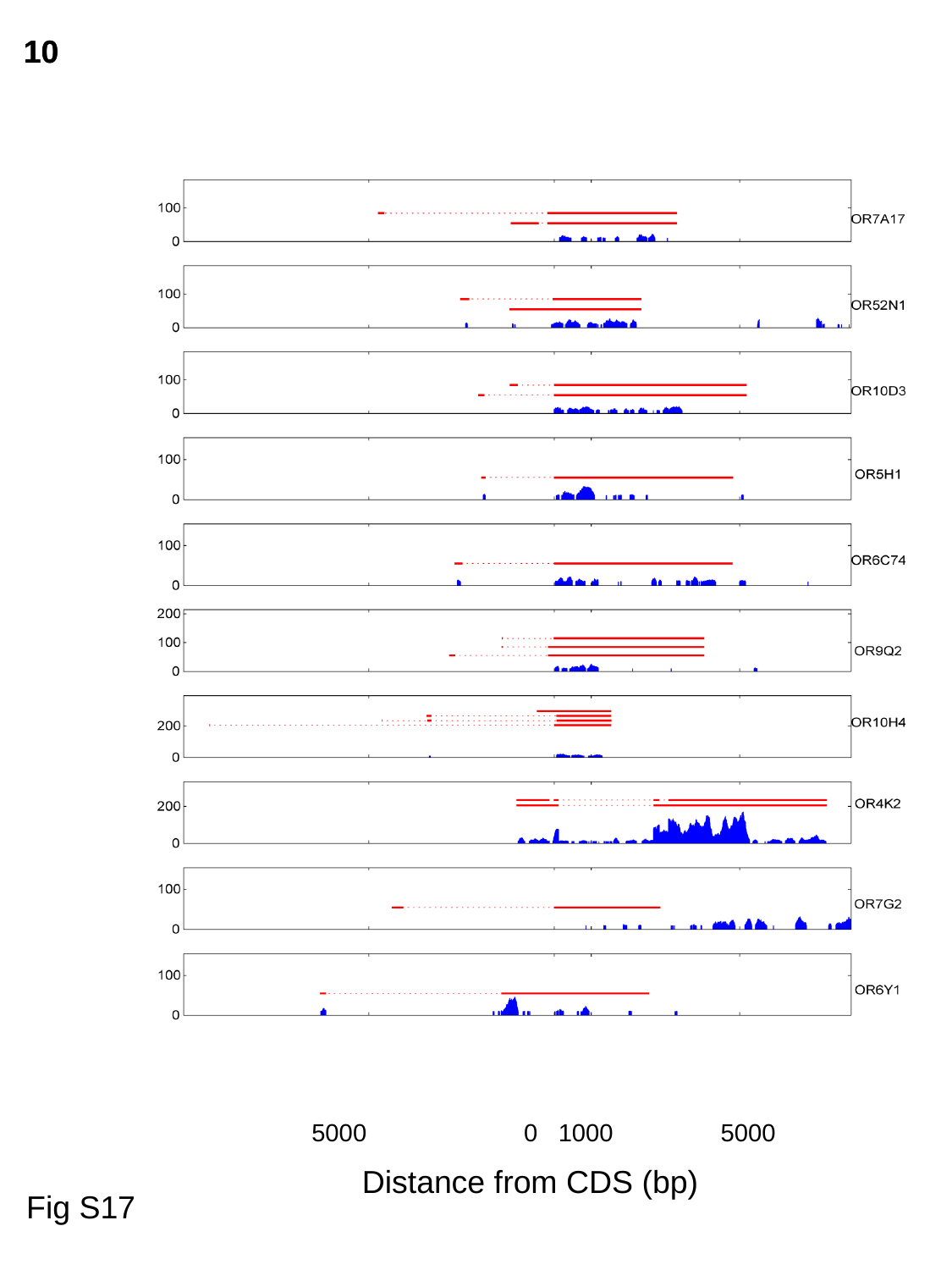

10
10
5000
0
1000
5000
Distance from CDS (bp)
Fig S17

## Slide 28
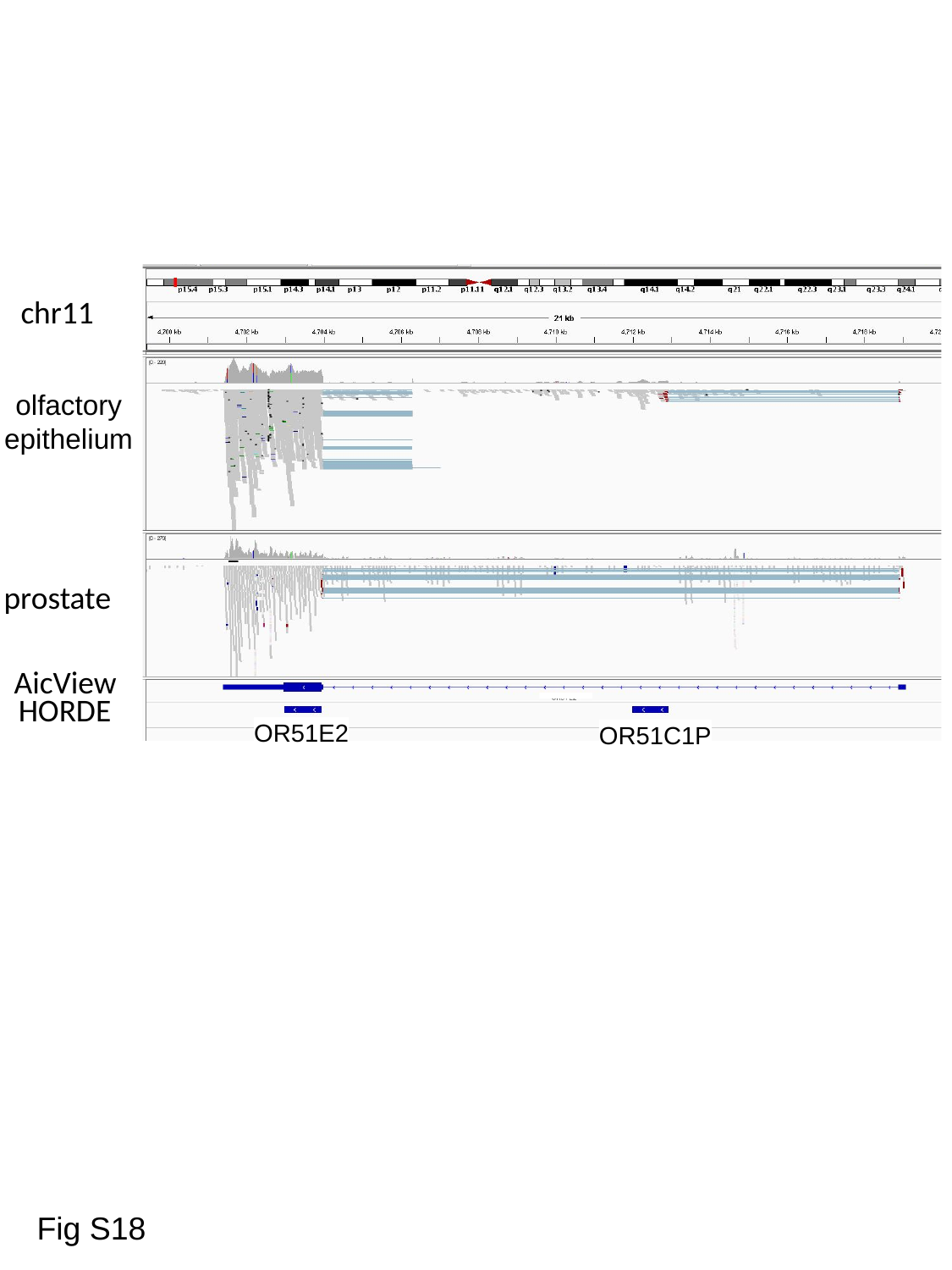

chr11
olfactory epithelium
prostate
AicView
HORDE
OR51E2
OR51C1P
Fig S18
